# Rational development of a human antibody cocktail that deploys multiple functions to confer Pan-SARS-CoVs protection

**DOI:** 10.1038/s41422-020-00444-y

**Published:** 2020-12-01

**Authors:** Hangping Yao, Yao Sun, Yong-Qiang Deng, Nan Wang, Yongcong Tan, Na-Na Zhang, Xiao-Feng Li, Chao Kong, Yan-Peng Xu, Qi Chen, Tian-Shu Cao, Hui Zhao, Xintian Yan, Lei Cao, Zhe Lv, Dandan Zhu, Rui Feng, Nanping Wu, Wenhai Zhang, Yuhao Hu, Keda Chen, Rong-Rong Zhang, Qingyu Lv, Shihui Sun, Yunhua Zhou, Run Yan, Guan Yang, Xinglu Sun, Chanjuan Liu, Xiangyun Lu, Linfang Cheng, Hongying Qiu, Xing-Yao Huang, Tianhao Weng, Danrong Shi, Weidong Jiang, Junbin Shao, Lei Wang, Jie Zhang, Tao Jiang, Guojun Lang, Cheng-Feng Qin, Lanjuan Li, Xiangxi Wang

**Affiliations:** 1 State Key Laboratory for Diagnosis and Treatment of Infectious Diseases/National Clinical Research Center for Infectious Diseases, First Affiliated Hospital, Zhejiang University School of Medicine, Hangzhou, Zhejiang 310003 China; 2 CAS Key Laboratory of Infection and Immunity, National Laboratory of Macromolecules, Institute of Biophysics, Chinese Academy of Sciences, Beijing, 100101 China; 3 State Key Laboratory of Pathogen and Biosecurity, Beijing Institute of Microbiology and Epidemiology, AMMS, Beijing, 100071 China; 4 Sanyou Biopharmaceuticals (Shanghai) Co., Ltd., Shanghai, 201114 China; 5 Shulan International Medical College, Zhejiang Shuren University, Hangzhou, Zhejiang 310015 China; 6 State Key Laboratory of Proteomics, Beijing Proteome Research Center, National Center for Protein Sciences (Beijing), Beijing Institute of Lifeomics, Beijing, 102206 China; 7 Shanghai Henlius Biotech, Inc, Shanghai, 200233 China; 8 Shanghai ZJ Bio-Tech Co., Ltd., Shanghai, 201114 China; 9 Guangzhou Regenerative Medicine and Health Guangdong Laboratory, Guangzhou, Guangdong 510200 China

**Keywords:** Structural biology, Immunology

## Abstract

Structural principles underlying the composition and synergistic mechanisms of protective monoclonal antibody cocktails are poorly defined. Here, we exploited antibody cooperativity to develop a therapeutic antibody cocktail against SARS-CoV-2. On the basis of our previously identified humanized cross-neutralizing antibody H014, we systematically analyzed a fully human naive antibody library and rationally identified a potent neutralizing antibody partner, P17, which confers effective protection in animal model. Cryo-EM studies dissected the nature of the P17 epitope, which is SARS-CoV-2 specific and distinctly different from that of H014. High-resolution structure of the SARS-CoV-2 spike in complex with H014 and P17, together with functional investigations revealed that in a two-antibody cocktail, synergistic neutralization was achieved by S1 shielding and conformational locking, thereby blocking receptor attachment and viral membrane fusion, conferring high potency as well as robustness against viral mutation escape. Furthermore, cluster analysis identified a hypothetical 3rd antibody partner for further reinforcing the cocktail as pan-SARS-CoVs therapeutics.

## Introduction

The ongoing pandemic of coronavirus disease 19 (COVID-19) has spurred an unprecedented public health crisis worldwide. To date, there have been more than 18 million confirmed cases of COVID-19 around the world, including about 700 thousand deaths according to the World Health Organization. The etiological agent of COVID-19 has been identified as a novel human coronavirus named severe acute respiratory syndrome coronavirus 2 (SARS-CoV-2).^1–3^ SARS-CoV-2 infection in humans results in a wide range of symptoms spanning from flu-like mild symptoms to more severe forms of pneumonia, acute respiratory distress syndrome, multiple organ failure, and even death. At present, there is no licensed vaccine or approved antiviral drug for treating COVID-19. Therefore new prophylactic and therapeutic strategies to combat human infections are urgently needed.

SARS-CoV-2 is an enveloped positive-sense, single-stranded RNA virus which belongs to the *betacoronavirus* genus within the *Coronaviridae* family that includes two other highly pathogenic coronaviruses, SARS-CoV and MERS-CoV. During the establishment of SARS-CoV-2 infection, the Spike (S) protein binds to its cellular receptor, angiotensin I converting enzyme 2 (ACE2) via the receptor binding domain (RBD). This is followed by a proteolytic cleavage step performed by host proteases, e.g., membrane serine protease, TMPRSS2, allowing the subsequent fusion of viral and host cellular membrane.^4^ Like other coronaviruses, the S consists of two subdomains: the N-terminal S1 domain, which possesses the N-terminal domain (NTD) and the C-terminal domain, also named as RBD, and the S2 domain, which is responsible for viral membrane fusion.^5^ Similar to other enveloped viruses (e.g., HIV), the S undergoes dramatic conformational changes from a prefusion to a postfusion state enabling fusion of viral and target cell membranes.^6^ Abrogation of this crucial role played by the S in the establishment of an infection is the main goal of neutralizing antibodies and the focus of therapeutic interventions.

A promising approach to combat the COVID-19 pandemic involves development of neutralizing antibodies (NAbs) against SARS-CoV-2. More recently, a panel of human antibodies with potent neutralizing activities against SARS-CoV-2 has been characterized and a few NAbs have been demonstrated to confer effective protection in animal models.^7–14^ However, when selective pressure is applied in immunotherapies, the emergence of viral mutations may result in resistance against antibodies, which has become a major concern. Such antibody/drug resistant mutants were firstly reported for HIV, which led to the development of the concept of cocktail therapies in order to overcome potential drug resistance encountered during the course of the therapy.^15^ Previous virological studies have revealed that the selection of antibodies binding to conserved epitopes, to some extent, mitigates mutational escape, but this strategy may not suffice. Furthermore, although antibodies targeting more conserved residues show cross-binding to multiple species of coronaviruses, they generally possess much weaker neutralizing activities than most NAbs specific for SARS-CoV-2.^10,12,16^ Therefore, a therapeutic antibody cocktail capable of targeting more conserved epitopes within the betacoronavirus genus as well as SARS-CoV-2-specific epitopes is likely to be more effective in the treatment of pan-betacoronavirus infections. More importantly, such a cocktail of antibodies might be more effective in conferring protection against escape mutants that may have developed resistance against an antibody.

## Results

### Identification and characterization of a candidate partner, NAb P17, for H014

**Fig. 1:**
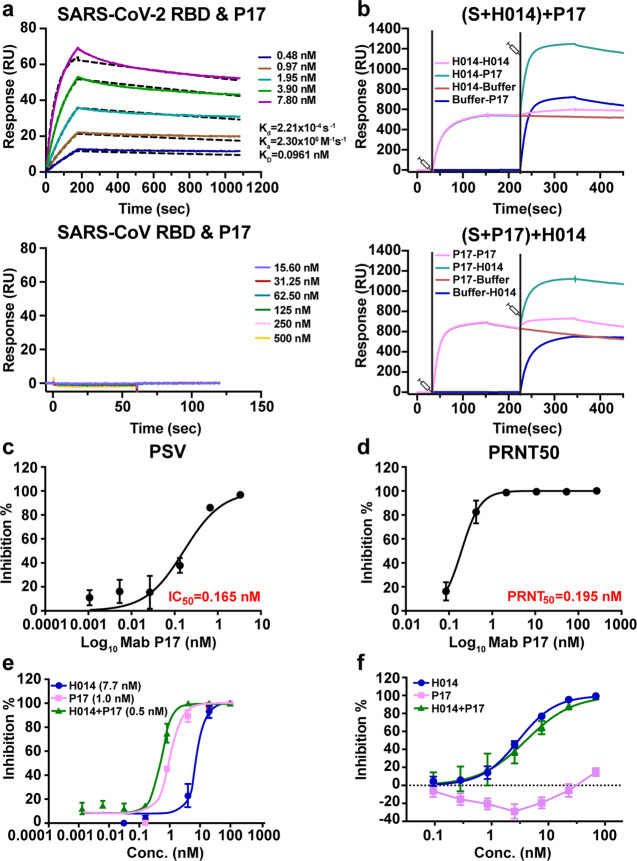
Characterization of a candidate partner, NAb P17, for H014. **a** Affinity analysis of the binding of P17 to SARS-CoV-2 RBD and SARS-CoV RBD. Purified SARS-CoV-2 RBD or the SARS-CoV RBD was immobilized onto a CM5 sensor chip surface and tested for real-time association and dissociation of the P17 antibody. **b** SPR kinetics of competitive binding of P17 and H014 to SARS-CoV-2 S. SARS-CoV-2 S was immobilized onto the sensor. H014 was first injected, followed by P17 (upper); vice-versa P17 was injected first and then H014 (lower). Pseudovirus neutralization test (PSV) of P17 in Huh7 cells (**c**) and in vitro neutralization activity of P17 against SARS-CoV-2 by PRNT in Vero cells (**d**). The data are from two independent experiments, values represent mean ± SD. Corresponding IC_50_ values are labeled. Neutralizing activities of P17, H014, and for the cocktail of antibodies at various concentrations (conc.) against SARS-CoV-2 live virus (**e**) and SARS-CoV pseudoviruses (**f**). Equimolar amounts of the antibodies were used in the assay.

By using phage display technique, we previously identified a humanized therapeutic antibody, H014, capable of cross-neutralizing SARS-CoV-2 and SARS-CoV via recognizing a conserved patch in the RBD.^12^ Monotherapy with H014 affords effective protection against a SARS-CoV-2 challenge in animal models.^12^ To select a partner for H014 for an ideal cocktail, we searched for antibodies that were (1) specific for SARS-CoV-2; (2) highly potent; (3) non-competing with H014 and RBD-targeting; (4) originating from humans. A large and diverse collection of antibodies were generated in the screening studies by using recombinant RBD of SARS-CoV-2 as a target against a fully human naive antibody library (ST-ST-HuNAL, Fab format, SanyouBio) using a standard solid-phase immunotube screening method. After 3 rounds of panning, a panel of more than 80 clones with unique sequences showing strong affinities against the target were selected as lead hits, and they were constructed into full-length IgG1 and expressed through the ExpiCHO system. Among the leads, the P17 antibody showed tight binding to SARS-CoV-2 RBD with a half maximal effective concentration (EC50) of 29 pM by the enzyme-linked immunosorbent assay (ELISA) (Supplementary information, Fig. S1). To investigate the viral specificity of P17, we expressed and purified recombinant SARS-CoV RBD for binding studies. Surface plasmon resonance (SPR) assays demonstrated that P17 bound to SARS-CoV-2 RBD with a high affinity of 96 pM. But it did not interact with SARS-CoV RBD, suggesting that P17 is SARS-CoV-2 specific (Fig. 1a). To gain insights into the epitopes recognized by P17, we used a competitive SPR-based epitope binding assay to map the antigenic sites present on the SARS-CoV-2 S trimer through H014, which binds a conserved epitope on one side of the RBD, distinct from the receptor binding motif (RBM).^12^ The CM5 sensor labeled with SARS-CoV-2 S trimer was saturated with H014 and flooded with P17 in the flow-through, or the sensor was first saturated with P17 followed by a flow of H014 over it. Although the S trimer was saturated with the first antibody (~600 RU for the binding signal), the second antibody could still bind to S trimer with the binding signal of up to 1200 RU. It indicated that P17 recognizes an epitope different from that bound by H014 (Fig. 1b), albeit without an allosteric binding mode for these two antibodies (Supplementary information, Fig. S2). We further assayed the in vitro neutralization capability of P17 against SARS-CoV-2. Immunofluorescence staining showed that P17 prevented SARS-CoV-2 infection in a dose-dependent manner in Vero cells (Supplementary information, Fig. S3). Pseudovirus neutralization assay (PSV) in Huh7 cells and standard 50% plaque reduction neutralization tests (PRNT) in Vero cells both showed that P17 exhibited stronger neutralizing activity with IC_50_ and PRNT_50_ values of 165 and 195 pM, respectively (Fig. 1c, d). Remarkably, the neutralizing activity of P17 against authentic SARS-CoV-2 is ~200-fold more potent than that of H014. Notably, a cocktail of P17 and H014 exhibited modest synergistic neutralization activity, even in the presence of a higher virus titer (Fig. 1e). Although P17 exhibited no inhibition activity against SARS-CoV in pseudotyping neutralization assay, the cocktail of P17 and H014 exhibited potent neutralizing activity against SARS-CoV pseudoviruses at sub-nM level due to the cross-neutralizing activity of H014, suggesting the potential of this cocktail to confer pan-SARS-CoVs protection (Fig. 1f).

### Prophylactic and therapeutic efficacy of P17 in SARS-CoV-2 susceptible mice

**Fig. 2:**
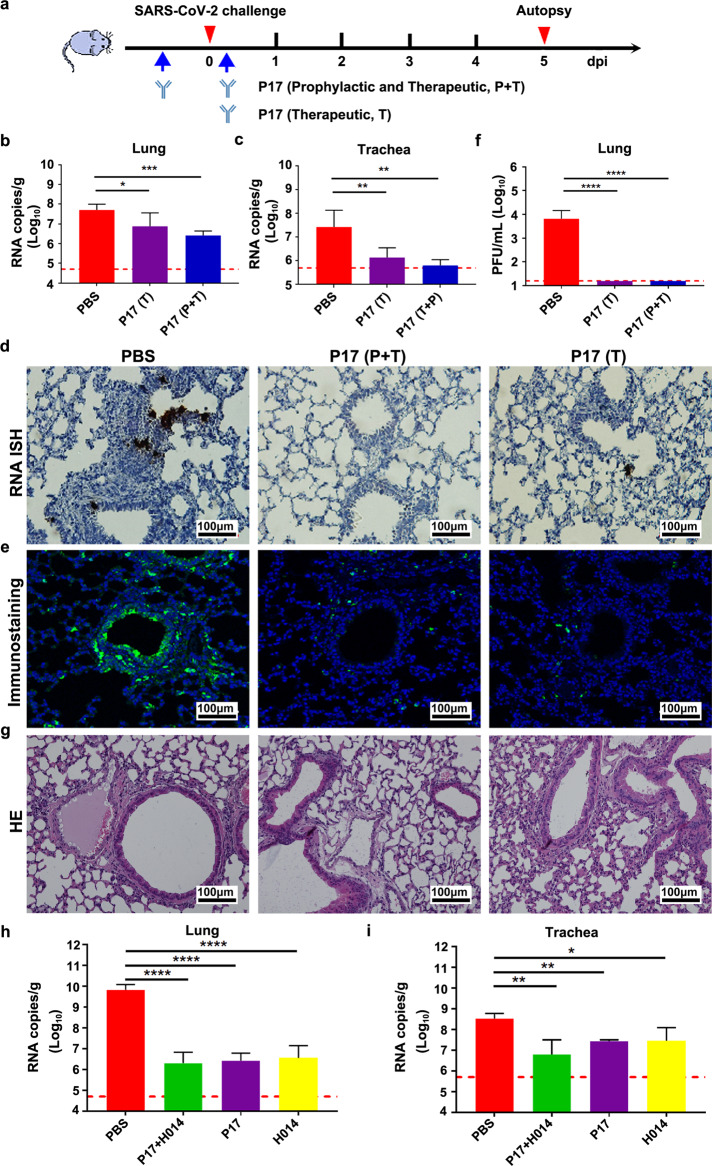
Prophylactic and therapeutic efficacy of P17. **a** Schematic diagram of P17 treatment in two SARS-CoV-2-susceptible mice models. Group of 6–8 week-old hACE2 mice and BALB/c mice were infected intranasally with 5 × 10^4^ PFU of SARS-CoV-2 or 1.6 × 10^4^ PFU of MASCp6 as described previously, respectively. Mice were treated in two independent experimental settings: (1) a single dose at 4 h post infection (Therapeutic, T); (2) two doses at 12 h before and 4 h post challenge (Prophylactic plus Therapeutic, P + T). **b** Virus RNA loads in the lungs were measured 5 dpi and are expressed as RNA copies per gram. *P < 0.05, **P < 0.01, and ***P < 0.005. **c** Virus RNA loads in the trachea were measured 5 dpi and are expressed as RNA copies per gram. **d** SARS-CoV-2 genome RNA ISH was performed with a SARS-CoV-2-specific probe. Brown-colored staining indicates positive results. Scale bar, 100 μm. **e** Immunostaining of mouse lung using SARS-CoV-2 spike-specific antibody. **f** Viral burden at 5 dpi in the lungs from the hACE2 mice, measured by plaque assay. Data are represented as mean ± SD. Dashed lines represent limit of detection. **g** Histopathological characterization of the lung from mice at 5 dpi. Scale bar, 100 μm. Therapeutic efficacy of this two-antibody cocktail in an established mouse model based on a SARS-CoV-2 mouse adapted strain MASCp6. Virus titers of lung (**h**) and trachea (**i**) tissues at 3 dpi determined by qRT-PCR (*P < 0.05, **P < 0.01, ***P < 0.005; ****P < 0.0001). Data are represented as means ± SD. Dashed lines represent limit of detection.

Given the excellent neutralizing activities of P17 at pM concentrations, we next sought to assess the in vivo protection efficacy of P17 in a newly established hACE2 mouse model.^17^ Intranasal inoculation of SARS-CoV-2 (5 × 10^4^ PFU) led to productive replication of the virus in the trachea and lungs of hACE2 mice. Mild pulmonary pathology was observed 5 days post inoculation (dpi). hACE2 mice were administered P17 12 h before or 4 h after inoculation of the SARS-CoV-2, representing a prophylactic (P) or therapeutic (T) setting, respectively (Fig. 2a). In the therapeutic mode, a single intraperitoneal injection of P17 at 20 mg per kilogram of body weight significantly reduced SARS-CoV-2 viral RNA loads in the lungs and trachea when compared to the control group (Fig. 2b, c). In the prophylactic mode, administration of two doses of P17 resulted in a more prominent effect, resulting in a 193-fold and 412-fold reduction of viral loads in the lungs and trachea, respectively (Fig. 2b, c). Compared to H014, a much lower administration dose (20 mg/kg vs 50 mg/kg) of P17 yielded a similar protective efficacy in reducing viral RNAs.^12^ Similarly, in situ hybridization (ISH) assays performed by RNAscope detected abundant SARS-CoV-2 specific RNAs in the control mice, while few viral RNA signals were detected in the P17-administered mice (Fig. 2d). Immunofluorescence staining of the lung section showed that only a few SARS-CoV-2 S protein-positive cells were detected upon P17 administration, while abundant viral protein expression was seen in alveolus along the airway in the PBS-treated mice (Fig. 2e). Notably, all mice from P17-treatment group no longer had infectious virus in the lung/trachea at 5 dpi as measured by a plaque assay of lung tissue homogenates (Fig. 2f). More importantly, histopathological examination revealed interstitial pneumonia, characterized by inflammatory cell infiltration, alveolar septal thickening and distinctive vascular system injury developed in hACE2 humanized mice belonging to the PBS control group at day 5 (Fig. 2g). In contrast, the lungs in mice from the P17-treated group only showed very mild inflammatory cell infiltration, and no obvious lesions of alveolar epithelial cells or focal hemorrhage (Fig. 2g). These results demonstrate that P17 is a potent neutralizing antibody, which is effective in conferring protection on mice against SARS-CoV-2. In addition, the cocktail of P17 and H014 exhibited 2–10-fold improvement on protective efficacy in an established mouse model based on a SARS-CoV-2 mouse adapted strain MASCp6 (Fig. 2h, i).

### Structures of the SARS-CoV-2 trimeric S in complex with P17

**Fig. 3:**
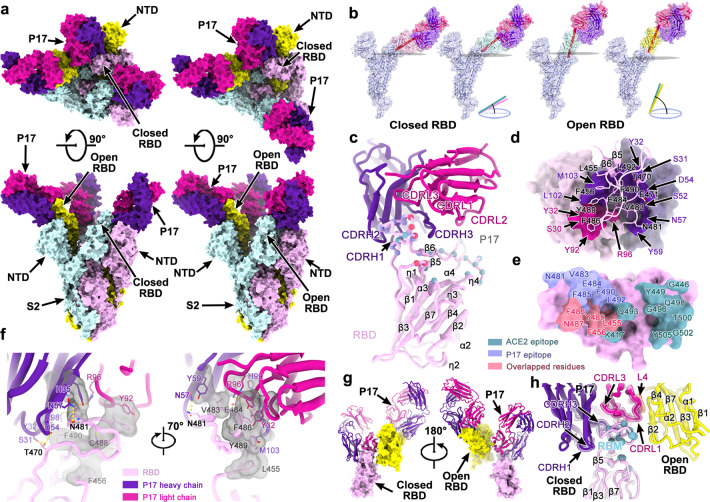
Cryo-EM structures of the SARS-CoV-2 S trimer in complex with P17. **a** Orthogonal views of the state 1 structure with one open and two closed RBDs (left) and the state 2 structure with two open and one closed RBDs (right). All the structures are presented as molecular surfaces with different colors for each S monomer (cyan, violet and yellow), and the P17 Fabs are shown in hotpink (light chains) and purpleblue (heavy chains). **b** Stochastic RBD rotations with different angles as a result of the switch between the “closed” and “open” states. **c** Cartoon representations of the structure of SARS-CoV-2 RBD (pink) in complex with P17 Fab. Residues comprising the P17 epitope and the RBM are shown as spheres and colored in blue and green, respectively. The overlapped residues between the P17 epitope and the RBM are shown in red. **d** The binding pocket of the P17 Fab and SARS-CoV-2 RBD. The epitope in SARS-CoV-2 RBD is presented as sticks, and the P17 is shown as surface. Detailed residues are indicated and the color scheme is the same as in **c**. **e** P17 epitope on SARS-CoV-2 RBD. The residues of P17 epitopes, ACE2-binding residues and residues binding to both P17 and ACE2 are colored in blue, green and red, respectively. **f** The interactions between the P17 Fab and SARS-CoV-2 RBD. Some residues involved in the formation of hydrophobic patches and hydrogen bonds are labeled and presented as sticks. **g** The P17 bound to the “closed” RBD also contacts RBD from its adjacent S. **h** Details of the interface between the P17 and SARS-CoV-2 RBD. Sites involved in interaction are labeled and L4 is emphasized by transparent orange tube.

To define the P17 epitope precisely, we characterized the complex between the P17 Fab fragments and a prefusion stabilized SARS-CoV-2 S ectodomain trimer using single-particle cryo-EM. Cryo-EM characterization of the complex revealed full occupancy for the antibody where one Fab is bound to each RBD of the homotrimeric S (Fig. 3a). Similar to previous studies,^18,19^ 3D classification revealed that the complex adopts two distinct conformational states, corresponding to one RBD open + two RBDs closed (state 1) and two RBDs open + one RBD closed (state 2) (Fig. 3a). We obtained asymmetric reconstructions at 3.6 and 3.8 Å resolution of the SARS-CoV-2–P17 complex in states 1 and 2, respectively (Supplementary information, Figs. S4–S7). The conformational mobility of the RBD-P17 region, representing a dynamic continuum of configurations, limited the map quality for the binding interface (Supplementary information, Fig. S5). To improve the resolution of the binding interface, local refinement by using an optimized “block-based” reconstruction approach^20–22^ was performed. This led to an improvement of the local resolution to 3.9 Å, enabling a reliable analysis of the interaction mode (Supplementary information, Figs. S5–S7 and Table S1).

P17 recognizes a conformational epitope on the receptor binding motif (RBM), only involving protein–protein contacts, which is consistent with the results of the competitive binding analysis performed in the presence of H014 (Fig. 1b). Theoretically, the glycosylation of SARS-CoV-2 S trimer may not affect the binding and protective efficacy of P17 and the cocktail of P17 and H014. Unlike the epitope of H014 that is accessible only when the RBDs are “open”, the P17 epitope is accessible in both the open and closed RBD states, which is in line with the stoichiometric binding of Fab to the S trimer (Fig. 3a; Supplementary information, Fig. S8). Expectedly, stochastic RBD rotations with different angles as a result of the switch between the “closed” and “open” states were observed in the complex structures (Fig. 3b; Supplementary information, Fig. S8). Tight bindings of P17 to various states of RBD by targeting the RBM suggest intense interferences during the whole dynamic interactions with host cells. The footprints of P17 cover an area of ~900 Å^2^ of one monomer of S. The variable domains of the light chain and heavy chain contribute ~30% and 70% to the interaction interface, respectively (Fig. 3c–e).

### The P17 epitope

Analysis of the structure of the complex of the P17 Fab bound with S helped define the epitope of the antibody. Interestingly, in addition to targeting the RBM, the P17 bound to the “closed” RBD contacts the neighboring RBD (residues 374–376 making up an upstream loop of β2) through the fourth solvent-exposed loop, the DE loop (named as L4 loop as well, adjacent to CDRL1 and CDRL2 that joins the D and E strands on the antibody), from the light chain (Fig. 3f–h; Supplementary information, Fig. S9). These contact residues on S may represent “non-essential” or “dynamic” epitopes for P17. Given the specific interaction mode involving forming contacts of two neighboring RBDs, P17 is capable of, to some extent, linking two adjacent S monomers, which probably restrains the conformational changes required for the progression of the life cycle of the virus from the prefusion to the postfusion stage (Fig. 3a, g).

P17 interacts with the RBD mainly through five complementarity-determining regions (CDR) loops: CDRL1 (residues 29–33), CDRL3 (residues 90–96), CDRH1 (residues 30–33), CDRH2 (residues 50–59) and CDRH3 (residues 99–106) (Fig. 3c, h; Supplementary information, Table S2). The essential P17 epitopes contains 14 residues that are located on the RBM (Supplementary information, Fig. S9). Amongst these, 11 residues (78.6%) are not conserved between SARS-CoV and SARS-CoV-2, explaining its virus-specific binding and neutralization activities (Supplementary information, Fig. S10). Similar epitopes identified in SARS-CoV by virus-specific NAbs, including m396, 80R and F26G19,^23–25^ together with the epitopes of SARS-CoV-2 revealed by P17 suggest that antibodies targeting the RBM core (residues 465–495 in SARS-CoV-2; residues 455–485 in SARS-CoV) primarily are more likely to be virus-specific. Tight binding is made possible by a network of hydrophobic interactions formed by L455, F456, V483, F486, C488, Y489 and F490 on the RBM, Y32, and Y92 of the light chain and Y32, H35, Y59, H99 and M103 of the heavy chain (Fig. 3d, f; Supplementary information, Table S2). In addition, extensive hydrophilic interactions further reinforce the RBD–P17 interaction. (Fig. 3d, f; Supplementary information, Table S2). More recently, a number of point mutations in the RBD have been reported in currently circulating strains. P17 exhibits comparable binding affinities to these RBD mutants (N354D/D364Y, V367F, R408I and W436R), indicative of a broad neutralizing potential of P17 against most circulating strains (Supplementary information, Figs. S10–S11).

### Synergistic neutralization mechanisms of P17 and H014

**Fig. 4:**
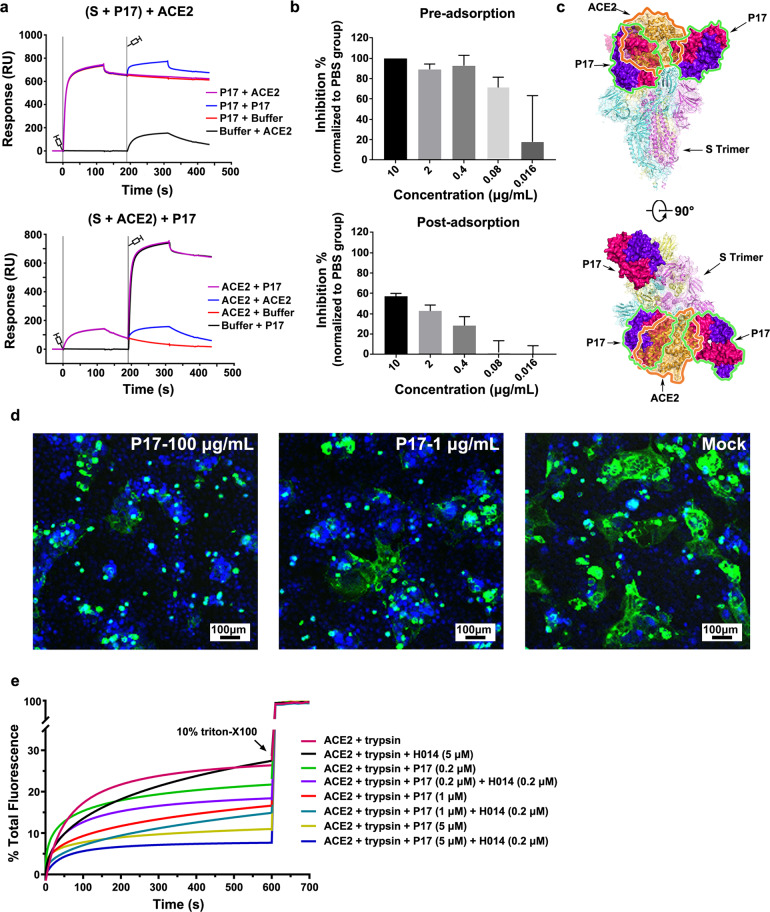
Synergistic neutralization mechanisms of P17 and H014. **a** SPR kinetics of competitive binding of P17 and ACE2 to SARS-CoV-2 S. SARS-CoV-2 S was immobilized onto the sensor. P17 was first injected, followed by ACE2 (upper), vice-versa ACE2 was injected first and then P17 (lower). The control groups are depicted by black curves. **b** Pre- and post-adsorption inhibition assays. Amounts of virus detected by RT-PCR when exposed to P17 before (upper) and after (lower) the virus was allowed to attach to cells. Values are mean ± SD. Experiments were repeated in duplicate. **c** Clashes between P17 Fab and ACE2 upon binding to SARS-CoV-2 S. P17 Fab is represented as surface; SARS-CoV-2 S trimer and ACE2 are shown as ribbon. **d** P17 inhibits S protein-mediated cell–cell fusion. 293T cells were transfected with SARS-CoV-2 S-GFP protein, co-cultured with Vero E6 cells in the absence or presence of 100 μg/mL or 1 μg/mL or 0 μg/mL P17. Images were taken after 48 h. Scale bar, 100 μm. **e** Different concentrations of P17 block the ACE2-mediated fusion of SARS-CoV-2 with liposomes. Low concentration of H014 has synergistic effects on P17 blocking. Liposomes were loaded with the fluorescent dye calcein with self-quenching concentrations. Fusion of SARS-CoV-2 with liposomes occurred in the presence of both ACE2 and trypsin, perturbing the bilayer resulting in the release of calcein and a consequent increase in fluorescence. 10% Triton X-100 treatment was used to achieve 100% calcein leakage. The data are from three independent experiments.

Previously, we have demonstrated that H014 neutralizes SARS-CoV-2 by preventing the attachment of the virus to its ACE2 receptor via steric clashes, albeit without targeting the RBM.^12^ In contrast to this, P17 binds to the RBM tightly and is therefore capable of blocking the binding of SARS CoV-2 to ACE2 completely by making the binding site unavailable. Results of the competitive inhibition assay demonstrated the abilities of P17 to effectively abrogate the interactions between SARS-CoV-2 RBD and ACE2 (Supplementary information, Fig. S12). Structural analysis revealed that the “closed” RBD conformation, corresponding to an ACE2-inaccessible state, is still capable of binding P17, indicative of a full occlusion of SARS-CoV-2 S trimer. To functionally verify this, two sets of SPR assays were performed by exposing the trimeric S to P17 first and then to ACE2 or the other way around. Expectedly, binding of P17 completely blocked the attachment of ACE2 to SARS-CoV-2 trimeric S and ACE2 that had already bound to trimeric S could be stripped and replaced by P17 owing to the substantially stronger binding affinity of P17 for SARS-CoV-2 trimeric S when compared to that of the ACE2 (Fig. 4a). To further verify these results in a cell-based viral infection model, pre- and post-adsorption inhibition assays were performed in Vero cells. As expected, incubation of P17 with SARS-CoV-2 prevented viral attachment to Vero cell surface in a dose-dependent manner and the viral particles that had already been adsorbed to the cell surface could be completely stripped by P17 at high concentrations (Fig. 4b). Superimposition of the structure of the SARS-CoV-2 S trimer-P17 complex over the structure of the SARS-CoV-2 RBD–ACE2 complex reveals severe clashes between ACE2 and its two adjacent P17, suggesting P17 would obstruct binding of the SARS-CoV-2 RBD to its ACE2 receptor (Fig. 4c). Due to the specific binding mode of P17, additional modes of neutralization may exist. To explore whether P17 could inhibit SARS-CoV-2 entry into host cells at the fusion stage, we established the S-mediated cell–cell fusion system using 293T cells that express SARS-CoV-2 S with a GFP tag as the effector cells and Vero-E6 cells as the target cells. After co-incubation of effector and target cells for 48 h, the SARS-CoV-2 S protein-expressing cells fused together into one large syncytium with multiple nuclei, while a pretreatment of effector cells with varying concentrations of P17 largely blocked the formation of the syncytium, suggesting that P17 is able to block viral membrane fusion (Fig. 4d). To further clarify the ability of P17 performed at the fusion stage, we established an in vitro membrane fusion assay where treatments of purified SARS-CoV-2 virions by trypsin and ACE2 could trigger viral membrane fusion with liposome at acidic environment. In line with cell–cell fusion assays, P17 could efficiently inhibit pH-dependent fusion of SARS-CoV-2 with liposomes in a dose-dependent manner, but H014 failed to do so even at a high concentration. Interestingly, P17 displays enhanced inhibition ability in presence of low concentration of H014, which is consistent with their synergistic neutralization activity (Figs. 4e and 1e).

### Cooperativity in cocktail of antibodies is achieved via S1 shielding and locking of conformation

**Fig. 5:**
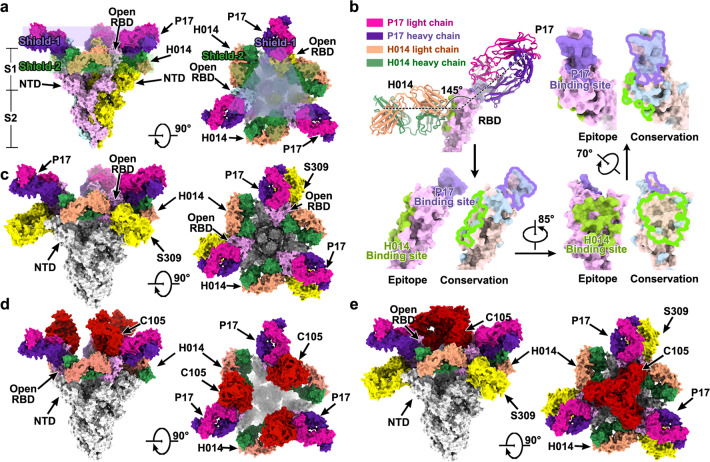
Structural basis for cooperativity in a cocktail consisting of P17 and H014 antibodies. **a** Overall structure of SARS-CoV-2 S trimer in complex with P17 and H014. 3 P17 Fabs bind at the top of each RBD, forming the “shield-1”; 3 H014 Fabs bind on the side of each RBD, constructing the “shield-2”. **b** The P17 and H014 bind distinct regions of the RBD. The P17 binding site is depicted in lightpurple and the H014 binding site is depicted in green. In the epitope conservation analysis between SARS-CoV-2 and SARS-CoV RBD, the epitope of P17 and H014 are silhouetted with lightpurple and green curve lines, respectively. The P17 binding site is not conserved between the RBDs of SARS-CoV-2 and SARS-CoV, whereas the H014 binding site is highly conserved between the SARS-CoV-2 and SARS-CoV RBDs. In the conservation analysis, wheat color represents high conservation and lightblue reflects low conservation. **c**–**e** Multiple potential cocktail candidates based on cocktail of P17 and H014 antibodies. SARS-CoV-2 S trimer is shown in gray. **c** Model of a hypothetical cocktail of antibodies consisting of P17 (hotpink and purpleblue), H014 (green and orange) and S309 (yellow). **d** Model of a cocktail of antibodies involving P17 (hotpink and purpleblue), H014 (green and orange) and C105 antibodies (red). **e** Model of a cocktail of antibodies consisting of P17 (hotpink and purpleblue), H014 (green and orange), S309 (yellow) and C105 (red) antibodies.

A synergistic effect was observed when H014 and P17 were used together for neutralizing SARS-CoV-2. To decipher the molecular basis for the synergetic effect of the two-antibody cocktail, we performed cryo-EM analysis of the SARS-CoV-2 S trimer in complex with H014 and P17 Fabs. An overall resolution of 3.2 Å could be attained for the cryo-EM structure of the ternary complex (Fig. 5a and Supplementary information, Fig. S6). Unlike most structural studies of the *apo* SARS-CoV-2 S trimer or its complexes where various conformational states corresponding to 0–3 RBDs open were observed,^10,12,18,19,26^ only one conformational state with all 3 RBDs open was observed in our structure of the S in complex with the cocktail of NAbs (Fig. 5a). This observation is also vastly different from the structural insights gained from the SARS-CoV-2 S in complex with H014 or P17 alone (Fig. 3). In total, there are 3 copies of the cocktail of NAb Fabs (3 H014 and 3 P17 Fabs) bound to one S trimer. While 3 P17 Fabs bind at the top of each RBD, overlapping and covering the contact regions of SARS-CoV-2 RBD with ACE2, forming a “cap” layer (shield-1), 3 H014 Fabs bind on the side of each RBD filling in the space between the RBD and its adjacent NTD, constructing an exterior layer (shield-2) with a staggered array beneath the cap layer (Fig. 5a). These two layers, acting as a shield, mask most of the regions of S1, preventing any possibility of the interaction of the SARS-CoV-2 RBD with host cellular receptor(s) and cell surface proteases (Fig. 5a). Furthermore, the 6 copies of Fabs work in synergy to prevent the closure of the RBD, restraining any further conformational changes in S1 that are required for initiating the viral fusion process. These structural insights explain the molecular basis for the mechanism of neutralization of SARS-CoV-2 by the cocktail of antibodies and provide a basis for rationalizing the cooperativity observed when both H014 and P17 are used for neutralizing SARS-CoV-2. The two NAbs of the cocktail can simultaneously bind to distinct regions of the RBD; one recognizing a more conserved patch across SARS-CoV and SARS-CoV-2 while the other targeting a SARS-CoV-2-specific patch (Fig. 5b), indicative of the potential of this cocktail in conferring a potent and broad protection against pan-SARS-CoV strains. In addition, a cocktail made from antibodies with non-competing activities, as in this cocktail, with at least one antibody recognizing highly conserved epitopes, theoretically confers additional protection against any eventuality of the virus escaping neutralization.

## Discussion

COVID-19 could be potentially treated more efficiently by combining potent neutralizing antibodies that target different epitopes. As the pandemic continues to spread through the population there is a possibility of emergence of mutants with abilities to escape antibody-mediated neutralization. A cocktail of antibodies could ensure protection against such mutants. A prospective goal is to identify highly potent neutralizing antibodies that could be combined into a broad therapeutic antibody cocktail, aiming to enhance protective potency as well as robustness to viral variants arising as the pandemic spreads or neutralization escape. A more recent study has concluded that the S2-directed antibodies and a large subset of NTD-directed antibodies exhibit no or very weak neutralizing activities.^9^ In addition, even within the group of the RBD-targeting NAbs, no strong correlation exists between the apparent binding affinity and neutralizing activity, suggesting the potency of an antibody appears to be largely determined by the nature and location of binding sites rather than, at least in part, just binding affinity.^9^ Consequently, it is important to decipher the immunogenic mechanism to discover and optimize a cocktail of antibodies that simultaneously recognize 2 or 3 different patches on the RBD. SPR-based cross-competition assays can provide information about clustering of antibodies on the target protein. However, it is important to bear in mind that the clusters identified by SPR do not necessarily equate to epitope clusters. In a recently published study, 3 clusters were identified on the RBD by performing cross-competition assays using soluble RBD instead of S trimer.^11^ We analyzed currently available structures of SARS-CoV-2 RBD-targeting NAbs to investigate whether a third partner, in line with the published cluster analysis,^11^ that together with H014 and P17 can bind simultaneously to the S trimer. S309, a cross-reactive NAb against SARS-CoV-2 and SARS-CoV recognizes a conserved glycan-containing epitope. Our structural analysis suggests that S309 can bind to this epitope and eschew steric clashes with H014 or P17, constituting the third neutralization cluster on the RBD of trimeric S^10^ (Fig. 5c). Complementarily, S309 performing neutralization via inducing antibody-dependent cell cytotoxicity (ADCC), but not involving the blocking of virus-receptor interaction,^10^ can be another ideal partner for our cocktail. Interestingly, another 3 NAbs (C105, B38 and CB6) targeting similar epitopes could also potentially work synergistically with H014 and P17 in a cocktail for binding to the RBD albeit with tiny clashes,^8,27,28^ possibly representing a fourth neutralization cluster on the RBD of trimeric S (Fig. 5d, e). However, this would require either of the 3 NAbs (C105, B38 and CB6) to slightly rotate inside to avoid steric clashes and thereby the S trimer can accommodate either of the 3 NAbs occupying the fourth cluster (C105 or B38 or CB6) together with 3 representative NAbs of a hypothetical cocktail (H014, P17 and S309) for simultaneously binding the RBD (Fig. 5d, e). Notably, a rotation towards the center of the S trimer may lead to new clashes raised by the other 2 copies of itself, probably further resulting in destruction of the S trimer, as observed in the neutralization mechanism for CR3022 antibody.^29^ Nevertheless, more experimental evidence is needed to verify the simultaneous binding of 4 NAbs to one RBD.

Herein we applied a rational NAb screening strategy coupled with SPR-based cross-competition assays and cryo-EM analysis to develop a next-generation human NAb cocktail, which can yield broad and potent protection against pan-SARS-CoVs. The cocktail of antibodies described in this study target spikes of SARS-CoV-2 and SARS-CoV, recognize the RBD at two distinct neutralization clusters and neutralize viral infections by blocking attachment to host cellular receptor as well as by interfering with viral membrane fusion, thereby conferring potency as well as additional protection against viral neutralization escape. Furthermore, our structural analysis related to the various neutralization clusters present on the RBD and the identification of additional putative antibody partner(s) is instructive for the development of an optimized therapeutic cocktail of antibodies for treating COVID-19. Principles of NAb-antigen interactions revealed here can also facilitate the design of therapeutic cocktails against other viruses.

## Materials and Methods

### Ethics statement

All animal studies were performed in strict accordance with the guidelines set by the Chinese Regulations of Laboratory Animals and Laboratory Animal-Requirements of Environment and Housing Facilities. All animal procedures were reviewed and approved by the Animal Experiment Committee of Laboratory Animal Center, AMMS, China (Assurance Number: IACUC-DWZX-2020-045).

### Cells and viruses

African green monkey kidney Vero (ATCC, #CCL-81) and Vero E6 cells were maintained in Dulbecco’s minimal essential medium (DMEM; Thermo Fisher Scientific) supplemented with 10% fetal bovine serum (FBS; Thermo Fisher Scientific), penicillin (100 U/mL) and streptomycin (100 μg/mL) (Thermo Fisher Scientific). SARS-CoV-2 were passaged in Vero cells and the virus stock was aliquoted and titrated to PFU/mL in Vero cells by plaque assay. All experiments involving infectious SARS-CoV-2 was performed under the biosafety level 3 facility.

### Protein expression and purification

The coding sequence for SARS-CoV-2 S trimer (residues 1–1208, GenBank: MN908947.3) was cloned into the mammalian expression vector pCAGGS with a C-terminal T4 fibritin trimerization motif, an HRV3C protease cleavage site and a 2× StrepTag to facilitate protein purification. The gene of S protein which was constructed with proline substitutions at residues 986 and 987 and a “GSAS” instead of “RRAR” at the furin cleavage sites was reported previously.^19^ The coding sequences for SARS-CoV RBD (residues 306–527, GenBank:NC_004718.3), SARS-CoV-2 RBD (residues 319–541, GenBank:MN908947.3) and the SARS-CoV-2 RBD mutants were cloned into the mammalian expression vector pCAGGS with a C-terminal his-tag. The expression vectors were used to transiently transfect HEK Expi 293F cells (Thermo Fisher Scientific) by polyethylenimine. Protein was purified from filtered cell supernatants using StrepTactin resin (IBA) or Ni-NTA resin (Qiagen) before being subjected to additional purification by size-exclusion chromatography in 20 mM Tris, 200 mM NaCl, pH 8.0.

### Screening and generation of humanized anti-SARS-CoV-2 antibody P17

Candidate antibody P17 was screened from phage-display fully human naive antibody library (simplified as ST-ST-HuNAL, constructed by Sanyoubio with the capacity of 2.2 × 10^11^, Fab format) by solid-phase immunotube screening method using recombinant RBD of SARS-CoV-2 as a target. The P17 antibody was prepared by ExpiCHO system (Thermo Fisher Scientific). Briefly, the plasmids harboring the heavy-chain and light-chain of the P17 antibody were mixed in ExpiFectamine^TM^ CHO, and then were added to the prepared cell suspension and mixed with gently shaking. The cell culture was then transferred and incubated at 37 °C in the shaker with 7% CO_2_. About 18–22 h after transfection, ExpiCHO^TM^ Enhancer and ExpiCHO^TM^ Feed were added and the cell culture was transferred to another shaker for incubation at 32 °C supplied with 5% CO_2_. Seven to fifteen days after transfection, the culture supernatant containing P17 antibody was collected and centrifuged at 12,000 × g for 10 min and the resulting supernatant was subjected to affinity purification with MabSelect SuRe LX (GE). P17 antibody was eluted with 100 mM Glycine-HCl (pH 3.0) and then neutralized with 1 M Tris-HCl. Finally, the purified antibody was exchanged into PBS buffer through the centrifugal filter unit (Millipore). Aliquots were made and stored in −80 °C.

### Surface plasmon resonance (SPR)

For the binding affinity assays, purified SARS-CoV-2 RBD and SARS-CoV RBD were immobilized onto a CM5 sensor chip surface using the NHS/EDC method and a PBS running buffer (supplemented with 0.05% Tween-20). Serial dilutions of purified P17 were injected. For the competitive binding assays, purified SARS-CoV-2 S was immobilized onto a CM5 sensor chip surface. The first sample flew over the chip at a rate of 20 μL/min for 120 s, then the second sample was injected at the same rate for another 120 s. For competitive binding SPR, The CM5 sensor labeled with SARS-CoV-2 S trimer was saturated with one antibody and flooded with the other antibody. In our cases, we indeed observed antibodies dissociated slightly (<10%) from S trimer and re-bound S trimer (to 100% from binding signals) when this antibody flowed again (Fig. 1b, H014–H014 and P17–P17), indicating that ~600 RU of binding signal represented a full occupancy for one binding site on S trimer. When the other antibody was applied, the RU value could reach up to 1200. Thus, it’s a reasonable analysis for simultaneous binding of H014 and P17 to S trimer.

### SARS-CoV-2 S pseudotyped virus neutralization

Construction of SARS-CoV-2 S pseudotyped virus (PSV) and PSV-based neutralization assay were performed as described previously.^30^ Briefly, threefold serial dilution antibody P17 were incubated with the same volume of SARS-CoV-2 pseudovirus with a TCID_50_ of 1.3 × 10^4^ for 1 h at 37 °C. The mixtures were then used to infect Huh7 cells seeded in 96-well plates for 24 h at 37 °C. After the incubation, supernatants were removed, and luciferase was added to each well and incubated for 2 min. After the incubation, luciferase activities were measured using a microplate spectrophotometer (PerkinElmer EnSight). The inhibition rate is calculated by comparing the OD value to the negative and positive control wells. IC_50_ were determined by a four-parameter logistic model using GraphPad Prism 7.0.

### PRNT

SARS-CoV-2 neutralization was measured using a standard PRNT assay as described previously. Briefly, fivefold serial dilution antibody P17 were added to ~100 PFU of SARS-CoV-2 and incubated for 1 h at 37 °C. For testing the neutralizing ability of P17 and H014 cocktail, fivefold serial diluted single mAb or mAb combination (equal molar ratio) were added to 500 PFU of SARS-CoV-2. Then, the mixture was added to Vero cell monolayers in a 24-well plate in duplicate and incubated for 1 h at 37 °C. The mixture was removed, and 1 mL of 1.0% (w/v) LMP agarose (Promega) in DMEM plus 4% (v/v) FBS was layered onto the infected cells. After further incubation at 37 °C for 2 days, the wells were stained with 1% (w/v) crystal violet dissolved in 4% (v/v) formaldehyde to visualize the plaques. Percentage of plaque reduction was calculated as:${\mathrm{Percentofplaquereduction}} = 100 - \frac{{{\mathrm{plaquenumberwithmAb}}}}{{{\mathrm{plaquenumberwithoutmAb}}}} \times 100$. The PRNT_50_ values were determined using non-linear regression analysis (GraphPad).

### In vivo treatment of P17 antibody in huACE2 mice infected with SARS-CoV-2

The in vivo protection efficacy of P17 antibody was tested in the established humanized ACE2 mice as described previously.^17^ Briefly, mice infected intranasally with 50 μL SARS-CoV-2 (5 × 10^4^ PFU) was subjected to P17 administration in prophylactic and/or therapeutic settings. In the therapeutic mode, a single dose of P17 (20 mg/kg) was injected intraperitoneally 4 h after SARS-CoV-2 challenge. In the prophylactic mode, two doses of P17 (20 mg/kg) were treated intraperitoneally at −12 h and 4 h after SARS-CoV-2 challenge. PBS injections were used as control treatments. The morbidity and mortality of the mice were recorded every day. Five days post infection, mice were sacrificed and the tissues of lung and trachea were collected for following-up viral burden determination (by RT-PCR) and plaque assay, pathological examination and immunofluorescence study.

### Viral burden determination

Viral burden in lung and trachea from mice were measured as described previously.^12^ Briefly, lung and trachea tissue homogenates were clarified by centrifugation at 6000× rpm for 6 min and viral RNA was extracted using the QIAamp Viral RNA Mini Kit (Qiagen) according to the manufacturer’s protocol. Viral burden in each tissue sample was performed by quantitative reverse transcription PCR (RT-qPCR) targeting the S gene of SARS-CoV-2. RT-qPCR was performed using One Step PrimeScript RT-PCR Kit (Takara).

### Histology, Immunostaining and RNA ISH

Lung tissues from mice were fixed with perfusion fixative (formaldehyde) for 48 h, and embedded in paraffin according to standard histological assays. For histopathology, lung tissues were stained with hematoxylin and eosin (H&E). Images were captured using Olympus BX51 microscope equipped with a DP72 camera. For immunostaining, paraffin tissue sections were deparaffinized with xylene, rehydrated through successive bathes of ethanol/water and incubated in 3% H_2_O_2_ at room temperature. The sections were then put in 10 mM sodium citrate buffer for 1 h at 96 °C for antigen retrieval and blocked with BSA at saturation for 20 min. Primary antibody was incubated for 2 h in a humidified chamber at 37 °C, followed by detection using the HRP-conjugated secondary antibody and TSA-dendronfluorophores. Afterwards, the primary and secondary antibodies were thoroughly eliminated by heating the slides in retrieval/elution buffer (Abcracker^®^, Histova Biotechnology, ABCFR5L) for 10 s at 95 °C using microwave. For RNA ISH assays were performed with an RNAscope 2.5 (Advanced Cell Diagnostics) according to the manufacturer’s instruction. Briefly, formalin-fixed paraffin-embedded tissue sections of 5 μm were deparaffinized by incubation for 60 min at 60 °C. Endogenous peroxidases were quenched with hydrogen peroxide for 10 min at room temperature. Slides were then boiled for 15 min in RNAscope Target Retrieval Reagents and incubated for 30 min in RNAscope Protease Plus before probe hybridization. The probe targeting SARS-COV-2 RNA was designed and synthesized by Advanced Cell Diagnostics (catalog no. 848561). Tissues were counterstained with Gill’s hematoxylin and visualized with standard bright-field microscopy. Original magnification was 20×.

### Pre- and post-adsorption inhibition assay

The pre- and post-adsorption inhibition assay of P17 were performed in Vero cells as previously described.^31–33^ In the post-adsorption assay, SARS-CoV-2 firstly were added to Vero cells for 1 h at 4 °C, then the cells were washed three times and P17 was added and incubated for additional 1 h at 4 °C. In the pre-adsorption assay, P17 was firstly incubated with SARS-CoV-2 for 1 h at 4 °C before viral infection. After three times washes with PBS, the PFU titers were calculated and the PRNT protocol was followed as described above.

### S-mediated cell-cell fusion system

The S-mediated cell-cell fusion assay was performed as previously described.^34,35^ Briefly, 293T cells transfected with SARS-CoV-2 S-GFP protein expression vectors were served as effector cells and Vero-E6 cells were used as target cells. Effector cells and target cells were co-cultured in the absence or presence of 1 μg/mL or 100 μg/mL P17 antibodies in DMEM containing 10% FBS for 48 h. After incubation, cells were fixed with 4% paraformaldehyde (PFA) at room temperature for 20 min and stained for nuclei with 4,6-diamidino-2-phenylindole (DAPI). The fluorescence images were recorded using a Leica SpeII confocal microscope. S-mediated cell-cell fusion was observed by the formation of multi-nucleated syncytia.

### Liposome fusion assay

Liposomes consisted of POPC, DOPS and Texas Red-DHPE were mixed and prepared as described previously.^36^ The dried lipid film was hydrated with 100 mM calcein (Sigma) in buffer (25 mM HEPES, 150 mM KCl, pH 7.4) at room temperature, and the vesicles were then extruded through filters (Whatman) with a pore size of 0.1 μm. Unincorporated calcein was separated from the liposomes using a Sephadex G-50 column. SARS-CoV-2 (~200 μg) was incubated with 0.1 μM trypsin (Sigma) at 37 °C for 20 min. Then the virus was mixed with 0.3 μM ACE2 and P17 antibody with the final concentration of 0.2, 1, 5 μM respectively with or without adding 0.2 μM H014 as a synergetic factor. The mixture was added to 0.1 mM liposomes in a 96-well plate, and the fluorescence (excitation at 460 nm, emission at 509 nm) was monitored at 37 °C using a SpectraMax M5 Microplate Reader (Molecular Devices). At *t* = 0 s, the pH of the medium was adjusted to 5.6 by addition of 10 μL of 1 M MES (pH 5.6) as *F*_0_. The emission fluorescence was recorded as *F*_t_ at 10 s intervals. After 600 s, 10 μL of 10% Triton X-100 was added to achieve complete release of the maximum fluorescence as *F*_100_. The fusion scale was calibrated such that 0% fusion corresponded to the initial excimer fluorescence value. The percentage of calcein leakage at each time point is defined as: leakage (%) = (*F*_t_ − *F*_0_) × 100/(*F*_100_ − *F*_0_).

### Production of Fab fragments for structural analysis

The P17 Fab fragments were generated using a Pierce^TM^ Fab Preparation Kit (Thermo Fisher Scientific), according to the manufacturer′s instructions. Briefly, antibody was mixed with papain and then digested at 37 °C for 4 h after removing the salt using a desalination column. The Fab fragment was separated from the Fc fragment by using a Hitrap Q column (GE Healthcare). Then, P17 Fab was collected and concentrated for cryo-EM analysis. The production of H014 Fab fragments were reported previously.^12^

### Negative stain

Samples to be examined were deposited onto a freshly glow-discharged carbon-coated grid for 1 min. Excess sample was removed and the grid was washed twice with buffer (20 mM Tris, 200 mM NaCl, pH 8.0) after which the sample was immediately negatively stained for 1 min with 1% phosphotungstic acid (pH 7.0). Excess stain was removed and the grid was loaded onto a 120 kV TEM for examination.

### Cryo-EM sample preparation and data collection

For cryo-EM sample preparation of SARS-CoV-2 S trimer–P17 complex, purified P17 Fab fragments were incubated with purified S trimer at a ratio of 9 Fab molecules per S trimer. For cryo-EM sample preparation of SARS-CoV-2 S trimer in complex with P17 and H014, purified P17 Fab fragments and H014 Fab fragments were incubated together with purified S trimer at a ratio of 9 P17 Fab molecules and 9 H014 Fab molecules per S trimer. A 3 μL aliquot of the mixture was transferred onto a freshly glow-discharged C-flat 1.2/1.3 Au grid. Grids were blotted for 3 s in 100% relative humidity for plunge-freezing (Vitrobot; Thermo Fisher Scientific) in liquid ethane. Cryo-EM datasets were collected at 300 kV using a Titan Krios microscope (Thermo Fisher Scientific) equipped with a K2 detector (Gatan, Pleasanton, CA). Movies (32 frames, each 0.2 s, total dose 60 e^−^Å^−2^) were recorded with a defocus of between 1.5 and 2.7 μm using SerialEM, which yields a final pixel size of 1.04 Å.

### Image processing, three-dimensional reconstruction, model building and refinement

For the complex of SARS-CoV-2 S trimer-P17, 4243 movies were recorded. The micrographs from each movie were aligned and averaged for the correction of beam-induced drift using MOTIONCOR2.^37^ The contrast transfer function parameters for each micrograph were estimated by Gctf.^38^ Particles from micrographs were picked automatically and extracted. A total of 450,514 particles were used to two-dimensional classification and three-dimensional classification. Two major classes (state 1 150,376 particles and state 2 98,932 particles) are picked out and used for further high-resolution refinement. After the high-resolution refinement and postprocessing (estimate the B-factor automatically), the final density map of state 1 and state 2 at 3.6 and 3.8 Å were obtained and evaluated on the basis of the gold-standard Fourier shell correlation (threshold = 0.143),^39^ respectively. Furthermore, all particles from state 1 and state 2 are used to do a “block-based” local refinement to improve P17 binding interface. The reconstruction at 3.9 Å was obtained after the local refinement and postprocess.

For the reconstruction of cocktail, 2831 movies were recorded. The micrographs from each movie were aligned and averaged for the correction of beam-induced drift using MOTIONCOR2. The contrast transfer function parameters for each micrograph were estimated by Gctf. Particles from micrographs were picked automatically and extracted. A total of 363,194 particles were used to two-dimensional classification and three-dimensional classification. Totally 175,063 particles are picked out and used for further high-resolution refinement with C1 symmetry. The relion_align_symmetry was used to generate the C3 reference giving rise to a better reconstruction (Supplementary information, Fig. S5), which means that the C3 symmetry could be imposed to improve resolution. After the high-resolution refinement and postprocessing (estimate the B-factor automatically), the final density map at 3.2 Å was obtained and evaluated on the basis of the gold-standard Fourier shell correlation (threshold = 0.143).

All procedures above were performed using Relion.^37^ The local resolution was evaluated by ResMap.^40^ The structures of the SARS-CoV-2 S trimer and human Fab fragments (Protein Data Bank ID: 6VSB, 5N4J and 7CAC) were manually fitted into the refined EM maps of SARS-CoV-2 S trimer-P17 complex and cocktail using the program Chimera^41^ and further corrected manually by real-space refinement in COOT. The atomic model was further refined by positional and B-factor refinement in real space using Phenix^42^ and rebuilding in COOT iteratively. The final models were evaluated by Molprobity.^43^ The datasets and refinement statistics are summarized in Supplementary information, Table S1.

### ELISA binding tests of P17 to Spike RBD and mutants

SARS-CoV-2 Spike RBD-his protein or mutants was coated in 96-well plate at 4 °C overnight. The 96-well plate was then blocked with PBS containing 5% non-fat milk powder for 1 h. Serial dilutions of P17 antibody were added into the plate and incubated for 2 h at room temperature. After washing away the excess antibodies, the HRP-labeled goat anti human Fc secondary antibody (1:5000, Abcam) was added and incubated for another 1 h. The plate was washed by PBST (supplied with 0.1% Tween-20), and then incubated with HRP substrate TMB for color development and quenched by adding 40 μL 2 M HCl. Finally, the binding ability was revealed by the absorbance at 450 nM wavelength using the plate reader (SurModics).

### Immunofluorescence of SARS-CoV-2 infection in Vero E6 cells after P17 treatment

A serial dilutions of P17 antibodies were prepared at 50 μL/well in a 96-well tissue culture plate in MEM (minimum essential medium) supplemented with 2 mM glutamine, 100 U/mL penicillin and 100 μg/μL streptomycin. An equal volume of SARS-CoV-2 (GISAID ID: EPI_ISL_415711) containing 100 TCID_50_ was added into the antibody-containing plate, mixed well and incubated for 60 min at room temperature. Meanwhile, Vero E6 cells were prepared at a concentration of 2 × 10^5^ cells/mL in MEM (minimum essential medium) supplemented with 2 mM glutamine, 100 U/mL penicillin, 100 μg/μL streptomycin and 5% FBS. Then 100 μL of cells (2 × 10^4^ cells per well) were transferred into the plate containing virus/antibody mixtures, mixed well and incubated at 37 °C and 5% CO_2_ in the cell incubator. After 4 days, culture medium supernatant containing the virus and antibody was collected and discarded. Pre-chilled acetone solution (80% in PBS) at 50 μL/well was added and incubated for 10 min. Then the plate was washed with 1× PBS twice, and the cells were blocked by 1% BSA in PBS for 30 min. After blocking buffer was discarded, anti-SARS-CoV-2 Spike protein antibody (1:1000, Sinobio) was added and incubated for another 90 min at room temperature. Then the plate was washed with 1× PBS for 3 times, and Alexa Fluor488^®^-conjugated Goat anti-rabbit IgG secondary antibody (Abcam) was added and incubated for 1 h at room temperature in dark. Cells were washed 3 times with 1× PBS and stained with 0.5 µg/mL DAPI for nuclei staining. Finally, after washing for 3 times with 1× PBS, immunofluorescence signals were obtained and imaged using a fluorescence microscope (BioRad ZOE).

### FACS blocking assay of antigen-receptor interaction by P17

Vero E6 cells validated for their confluence and viability were detached by trypsin-EDTA. After centrifugation at 4 °C, 300× *g* for 2 min, the cells were resuspended in FACS buffer (1× PBS + 2% FBS) at a density of 2 × 10^6^ cells/mL, and 100 μL of the cells were added into each well of a 96-well round bottom plate. Meanwhile, in another 96-well plate, serial dilutions of P17 were mixed to the equal volume of 2 μg/mL RBD-mFc. After incubation at 4 °C for 1 h, the mixture was transferred to the plate containing Vero E6 cells, mixed well and incubated at 4 °C for 0.5 h. Then the plate was subjected to centrifugation at 4 °C, 300× *g* for 2 min, and the cells were resuspended and washed by FACS buffer for three times. Then PE labeled goat anti-mouse Fc secondary antibody (1:200, Jackson) was added into the plate and incubated at 4 °C for 0.5 h. After centrifugation at 4 °C, 300× *g* for 2 min, the cells were resuspended and washed by FACS buffer for three times. Finally, the binding of RBD-mFc on the cells were detected and analyzed by flow cytometry (Beckman, CytoFLEX, AOO-1-1102).

## Supplementary information

Supplementary Figure S1

Supplementary Figure S2

Supplementary Figure S3

Supplementary Figure S4

Supplementary Figure S5

Supplementary Figure S6

Supplementary Figure S7

Supplementary Figure S8

Supplementary Figure S9

Supplementary Figure S10

Supplementary Figure S11

Supplementary Figure S12

Supplementary Table S1

Supplementary Table S2

## Data Availability

Cryo-EM density maps of the SARS-CoV-2 S trimer in complex with P17 (state 1), SARS-CoV-2 S trimer in complex with P17 (state 2), SARS-CoV-2 S trimer in complex with P17 and H014 cocktail and binding interface between P17 and the RBD have been deposited at the Electron Microscopy Data Bank with accession codes EMD-30483, EMD-30482, EMD-30484 and EMD-30485 and the related atomic models have been deposited in the Protein Data Bank under accession code 7CWM, 7CWL, 7CWN and 7CWO, respectively.

## References

[1] Zhou P (2020). A pneumonia outbreak associated with a new coronavirus of probable bat origin. Nature.

[2] Wu F (2020). A new coronavirus associated with human respiratory disease in China. Nature.

[3] Zhu N (2020). A Novel Coronavirus from Patients with Pneumonia in China, 2019. N. Engl. J. Med..

[4] Hoffmann M (2020). SARS-CoV-2 cell entry depends on ACE2 and TMPRSS2 and is blocked by a clinically proven protease inhibitor. Cell.

[5] Kirchdoerfer RN (2016). Pre-fusion structure of a human coronavirus spike protein. Nature.

[6] Li F (2016). Structure, function, and evolution of coronavirus spike proteins. Annu. Rev. Virol..

[7] Rogers TF (2020). Isolation of potent SARS-CoV-2 neutralizing antibodies and protection from disease in a small animal model. Science.

[8] Shi R (2020). A human neutralizing antibody targets the receptor binding site of SARS-CoV-2.. Nature.

[9] Wec AZ (2020). Broad neutralization of SARS-related viruses by human monoclonal antibodies. Science.

[10] Pinto D (2020). Cross-neutralization of SARS-CoV-2 by a human monoclonal SARS-CoV antibody. Nature.

[11] Brouwer PJM (2020). Potent neutralizing antibodies from COVID-19 patients define multiple targets of vulnerability. Science.

[12] Lv Z (2020). Structural basis for neutralization of SARS-CoV-2 and SARS-CoV by a potent therapeutic antibody. Science.

[13] Zhang, L. et al. A proof of concept for neutralizing antibody-guided vaccine design against SARS-CoV-2. *bioRxiv*10.1101/2020.09.23.309294 (2020). 10.1093/nsr/nwab053 PMC8083607 34676098

[14] Wang, N. et al. Structure-based development of human antibody cocktails against SARS-CoV-2. *Cell Res.*10.1038/s41422-020-00446-w (2020). 10.1038/s41422-020-00446-w PMC7705432 33262454

[15] Awi NJ, Teow SY (2018). Antibody-Mediated Therapy against HIV/AIDS: Where Are We Standing Now?. J. Pathog..

[16] Baum A (2020). Antibody cocktail to SARS-CoV-2 spike protein prevents rapid mutational escape seen with individual antibodies. Science.

[17] Sun SH (2020). A mouse model of SARS-CoV-2 infection and pathogenesis. Cell Host Microbe.

[18] Walls AC (2020). Structure, function, and antigenicity of the SARS-CoV-2 spike glycoprotein. Cell.

[19] Wrapp D (2020). Cryo-EM structure of the 2019-nCoV spike in the prefusion conformation. Science.

[20] Wang N (2019). Architecture of African swine fever virus and implications for viral assembly. Science.

[21] Yang Y (2020). Architecture of the herpesvirus genome-packaging complex and implications for DNA translocation. Protein Cell.

[22] Wang N (2020). Structures of the portal vertex reveal essential protein-protein interactions for Herpesvirus assembly and maturation. Protein Cell.

[23] Hwang WC (2006). Structural basis of neutralization by a human anti-severe acute respiratory syndrome spike protein antibody, 80R. J. Biol. Chem..

[24] Prabakaran P (2006). Structure of severe acute respiratory syndrome coronavirus receptor-binding domain complexed with neutralizing antibody. J. Biol. Chem..

[25] van den Brink EN (2005). Molecular and biological characterization of human monoclonal antibodies binding to the spike and nucleocapsid proteins of severe acute respiratory syndrome coronavirus. J. Virol..

[26] Xiong X (2020). A thermostable, closed SARS-CoV-2 spike protein trimer. Nature Struct. Mol. Biol..

[27] Wrapp D (2020). Structural basis for potent neutralization of Betacoronaviruses by single-domain Camelid antibodies. Cell.

[28] Barnes, C. O. et al. Structures of human antibodies bound to SARS-CoV-2 spike reveal common epitopes and recurrent features of antibodies. *Cell* **182**, 828–842 (2020). 10.1016/j.cell.2020.06.025 PMC7311918 32645326

[29] Huo, J. et al. Neutralization of SARS-CoV-2 by destruction of the prefusion spike. *Cell Host Microbe* **28**, 445–454 (2020). 10.1016/j.chom.2020.06.010 PMC7303615 32585135

[30] Nie J (2020). Establishment and validation of a pseudovirus neutralization assay for SARS-CoV-2. Emerg. Microbes Infect..

[31] Wang X (2017). Potent neutralization of hepatitis A virus reveals a receptor mimic mechanism and the receptor recognition site. Proc. Natl Acad. Sci. USA.

[32] Wang K (2020). Serotype specific epitopes identified by neutralizing antibodies underpin immunogenic differences in Enterovirus B. Nat. Commun..

[33] Wang K (2020). Structures of Echovirus 30 in complex with its receptors inform a rational prediction for enterovirus receptor usage. Nat. Commun..

[34] Xia S (2020). Inhibition of SARS-CoV-2 (previously 2019-nCoV) infection by a highly potent pan-coronavirus fusion inhibitor targeting its spike protein that harbors a high capacity to mediate membrane fusion. Cell Res..

[35] Zhu, L. et al. Double lock of a potent human therapeutic monoclonal antibody against SARS-CoV-2. *bioRxiv*10.1101/2020.11.24.393629 (2020). 10.1093/nsr/nwaa297 PMC7798916 34676096

[36] Qiu X (2018). Structural basis for neutralization of Japanese encephalitis virus by two potent therapeutic antibodies. Nat. Microbiol..

[37] Scheres SH (2016). Processing of structurally heterogeneous Cryo-EM data in RELION. Methods Enzymol..

[38] Zhang K (2016). Gctf: Real-time CTF determination and correction. J. Struct. Biol..

[39] Scheres SH, Chen S (2012). Prevention of overfitting in cryo-EM structure determination. Nat. Methods.

[40] Kucukelbir A, Sigworth FJ, Tagare HD (2014). Quantifying the local resolution of cryo-EM density maps. Nat. Methods.

[41] Yang Z (2012). UCSF Chimera, MODELLER, and IMP: an integrated modeling system. J. Struct. Biol..

[42] Afonine PV (2012). Towards automated crystallographic structure refinement with phenix.refine. Acta Crystallogr. Sect. D Biol. Crystallogr..

[43] Chen VB (2010). MolProbity: all-atom structure validation for macromolecular crystallography. Acta Crystallogr. Sect. D, Biol. Crystallogr..

