# Supplementary material for: Rational development of a human antibody cocktail that deploys multiple functions to confer Pan-SARS-CoVs protection
Source: Cell Res. 2020 Dec 1;31(1):25–36. doi: 10.1038/s41422-020-00444-y (PMC7705443; doi:10.1038/s41422-020-00444-y)
Supplement: Supplementary file 1 — Supplementary Figure S1 [file 41422_2020_444_MOESM1_ESM.pdf]

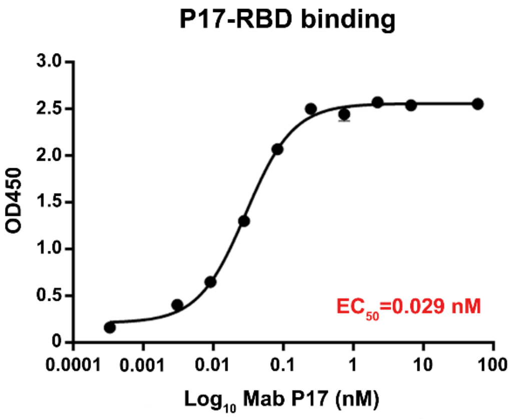

Fig. S1 Binding assay for recombinant RBD and P17 by ELISA. The P17 antibody showed strong binding to SARS-CoV-2 RBD with an EC50 value of 29 pM.
