# Supplementary material for: Rational development of a human antibody cocktail that deploys multiple functions to confer Pan-SARS-CoVs protection
Source: Cell Res. 2020 Dec 1;31(1):25–36. doi: 10.1038/s41422-020-00444-y (PMC7705443; doi:10.1038/s41422-020-00444-y)
Supplement: Supplementary file 2 — Supplementary Figure S2 [file 41422_2020_444_MOESM2_ESM.pdf]

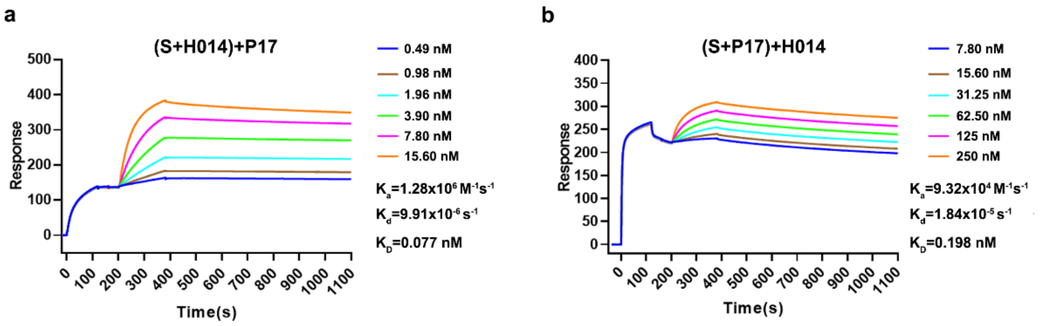

Fig. S2 Binding assays for S trimer with P17 or H014 after the first antibody was saturated by SPR. a, b SARS-CoV-2 S trimer was immobilized onto the sensor. H014 was first injected to saturation, followed by binding assays for P17 (a); vice-versa P17 was injected first and then H014 (b).
