# Supplementary material for: Rational development of a human antibody cocktail that deploys multiple functions to confer Pan-SARS-CoVs protection
Source: Cell Res. 2020 Dec 1;31(1):25–36. doi: 10.1038/s41422-020-00444-y (PMC7705443; doi:10.1038/s41422-020-00444-y)
Supplement: Supplementary file 3 — Supplementary Figure S3 [file 41422_2020_444_MOESM3_ESM.pdf]

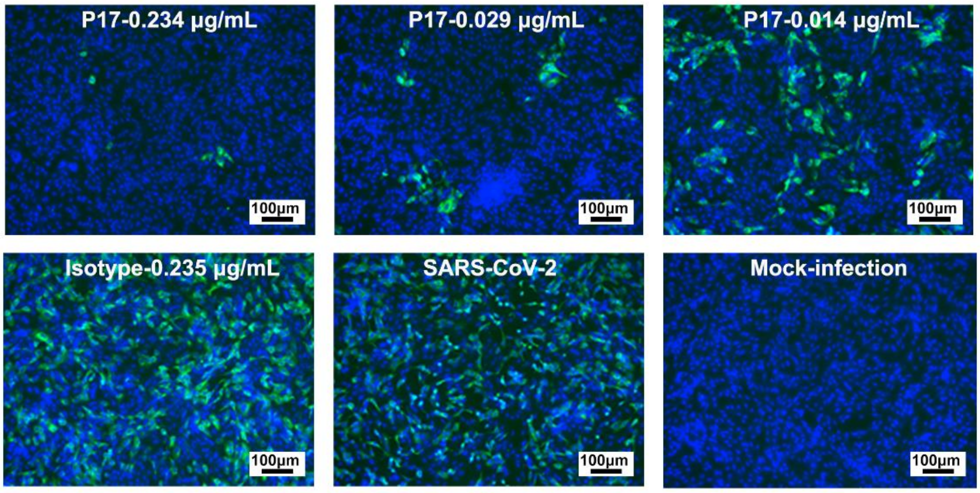

Fig. S3 Immunofluorescence of SARS-CoV-2 infection in Vero E6 cells after P17 treatment. Immunofluorescence staining showed that P17 prevented SARS-CoV-2 infection in a dose-dependent manner in Vero cells. Scale bar, 100 μm.
