# Supplementary material for: Rational development of a human antibody cocktail that deploys multiple functions to confer Pan-SARS-CoVs protection
Source: Cell Res. 2020 Dec 1;31(1):25–36. doi: 10.1038/s41422-020-00444-y (PMC7705443; doi:10.1038/s41422-020-00444-y)
Supplement: Supplementary file 4 — Supplementary Figure S4 [file 41422_2020_444_MOESM4_ESM.pdf]

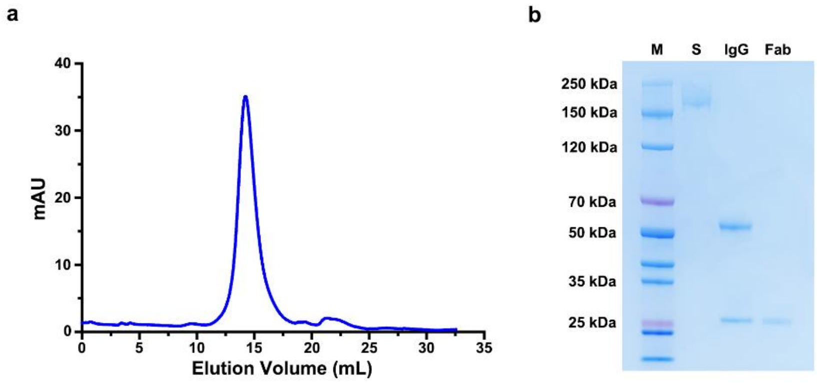

Fig. S4 Purification and characterization of SARS-CoV-2 S trimer and P17. a Gel filtration chromatogram of SARS-CoV-2 S trimer. Data from a Superose 6 10/300 column are shown in blue. **b** SDS-PAGE analysis of the purified SARS-CoV-2 S trimer, P17 IgG and P17 Fab.
