# Supplementary material for: Rational development of a human antibody cocktail that deploys multiple functions to confer Pan-SARS-CoVs protection
Source: Cell Res. 2020 Dec 1;31(1):25–36. doi: 10.1038/s41422-020-00444-y (PMC7705443; doi:10.1038/s41422-020-00444-y)
Supplement: Supplementary file 5 — Supplementary Figure S5 [file 41422_2020_444_MOESM5_ESM.pdf]

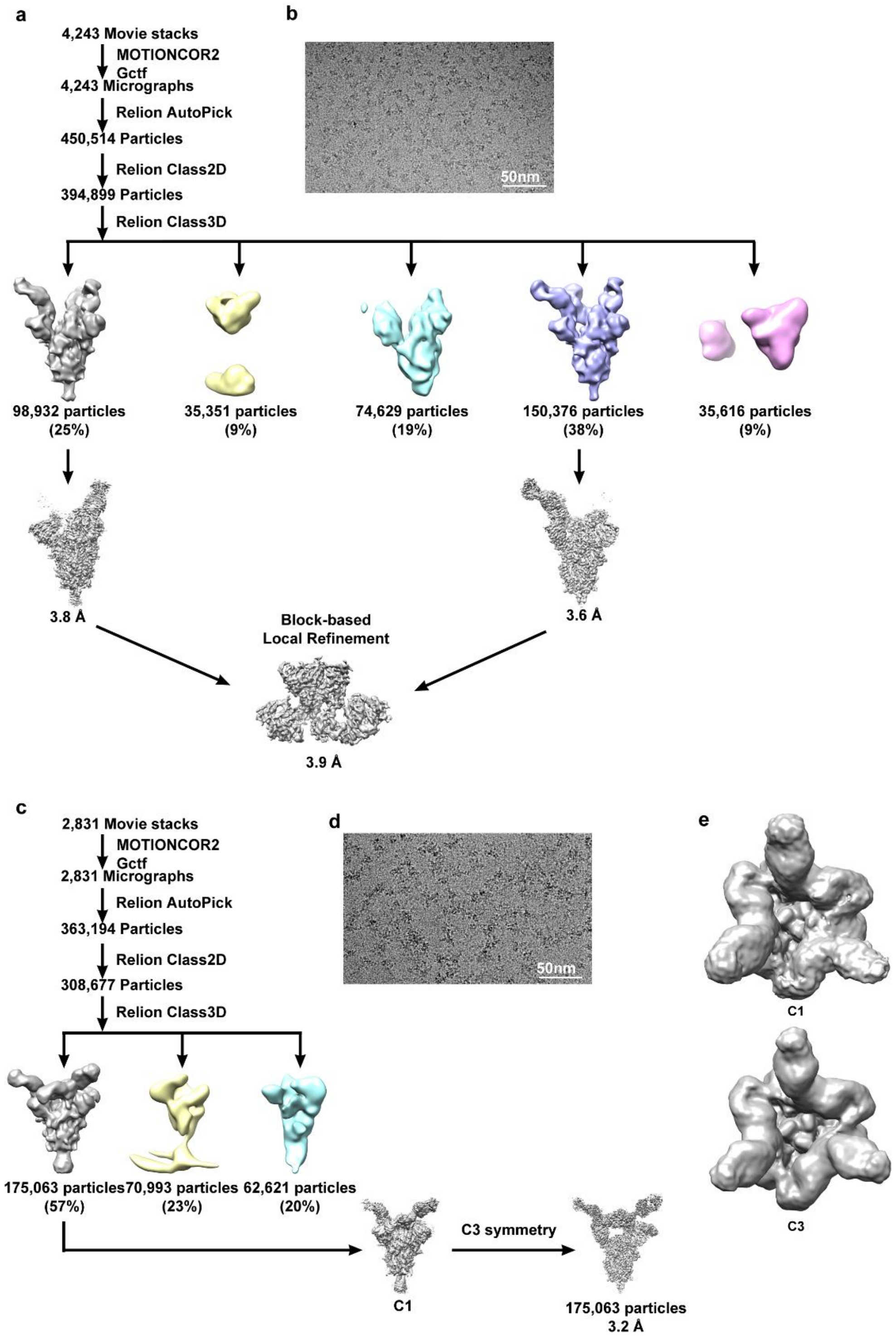

Fig. S5 Flow chart for cryo-EM data processing. **a** Flow chart for cryo-EM data processing of P17-Spike complex. **b** Cryo-EM image of P17-Spike complex. **c** Flow chart for cryo-EM data processing of P17-H014-Spike cocktail. **d** Cryo-EM image of P17-H014-Spike. **e** Structural comparison of P17-H014-Spike reconstructions by imposing C1 or C3 symmetry.
