# Supplementary material for: Rational development of a human antibody cocktail that deploys multiple functions to confer Pan-SARS-CoVs protection
Source: Cell Res. 2020 Dec 1;31(1):25–36. doi: 10.1038/s41422-020-00444-y (PMC7705443; doi:10.1038/s41422-020-00444-y)
Supplement: Supplementary file 6 — Supplementary Figure S6 [file 41422_2020_444_MOESM6_ESM.pdf]

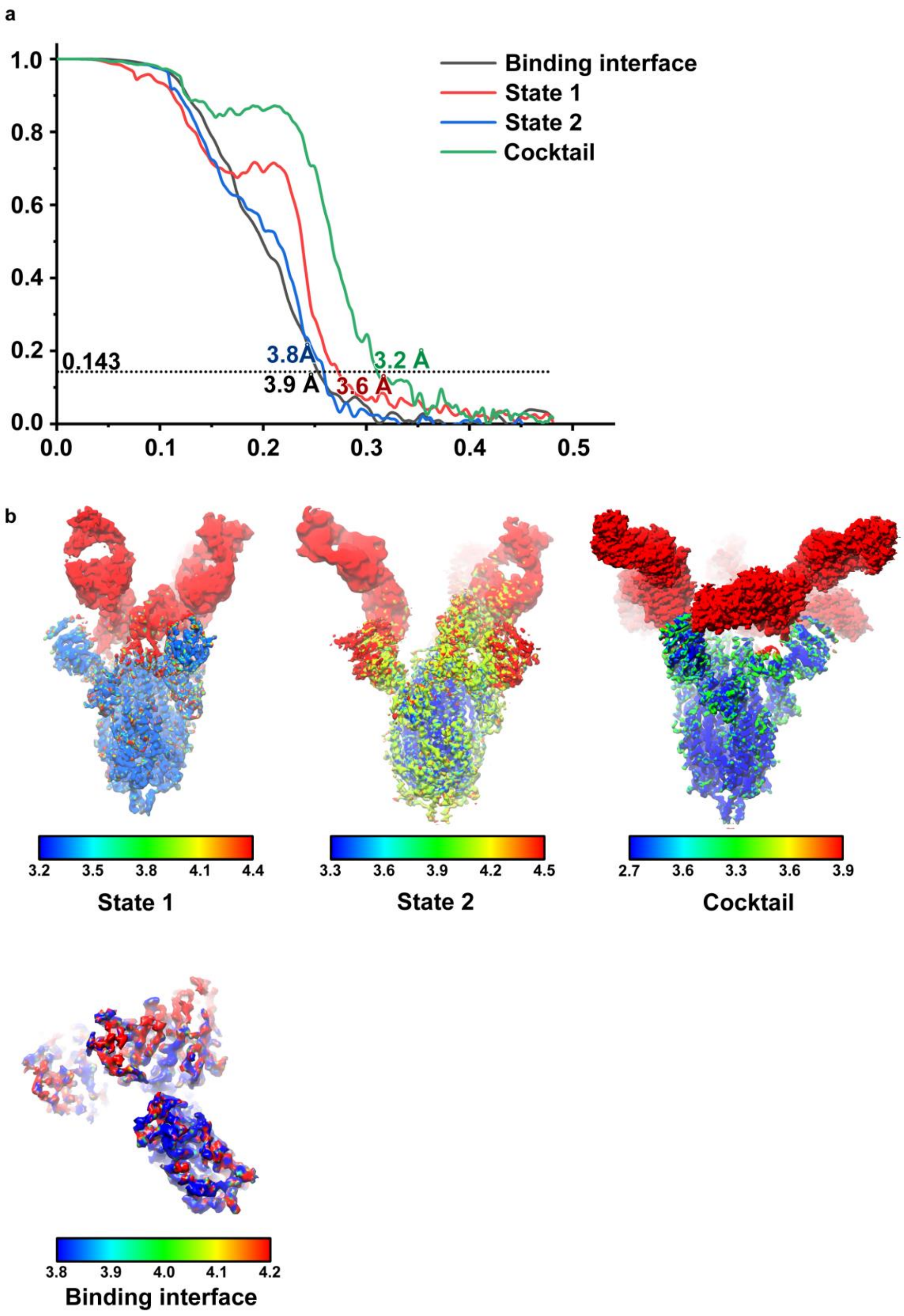

Fig. S6 Validation of the cryo-EM maps. a The gold-standard FSC curves of the final maps. **b** Local resolution of the cryo-EM maps of the P17-Spike state 1, state 2, cocktail and the binding interface evaluated using ResMap.
