# Supplementary figures and images for: Rational development of a human antibody cocktail that deploys multiple functions to confer Pan-SARS-CoVs protection
Source: Cell Res. 2020 Dec 1;31(1):25–36. doi: 10.1038/s41422-020-00444-y (PMC7705443; doi:10.1038/s41422-020-00444-y)

a

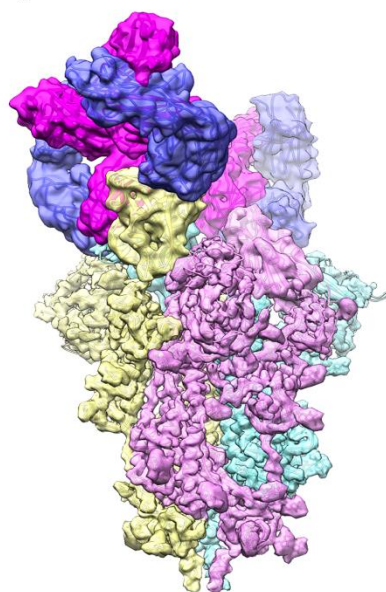

**State 1**

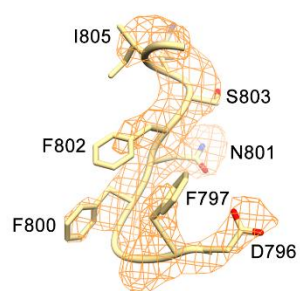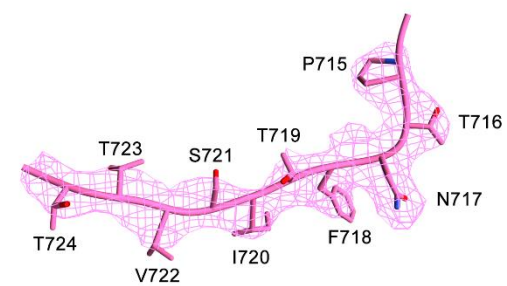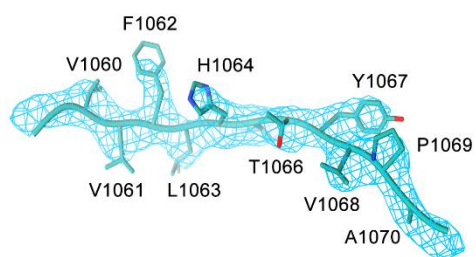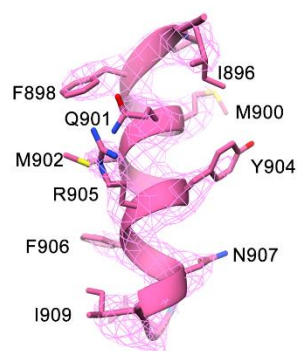

b

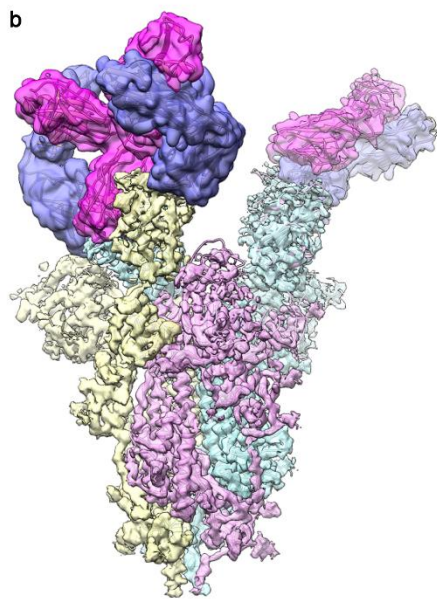

State 2

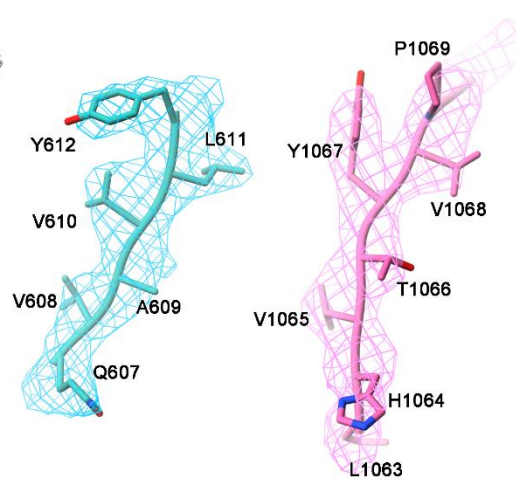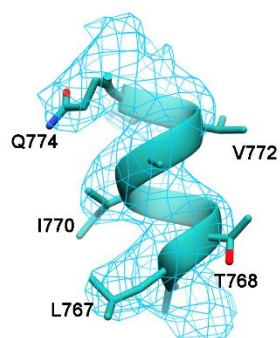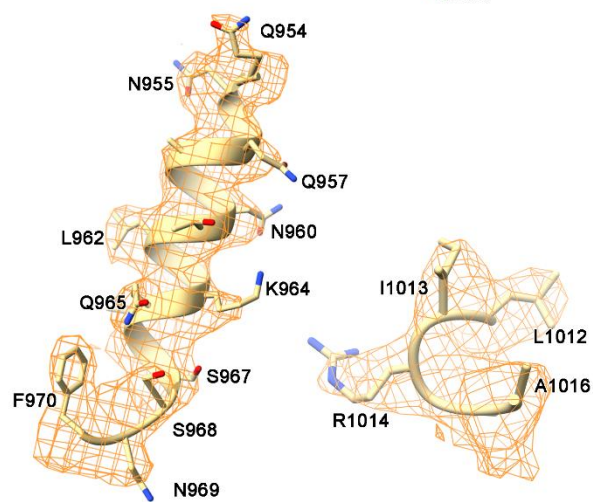

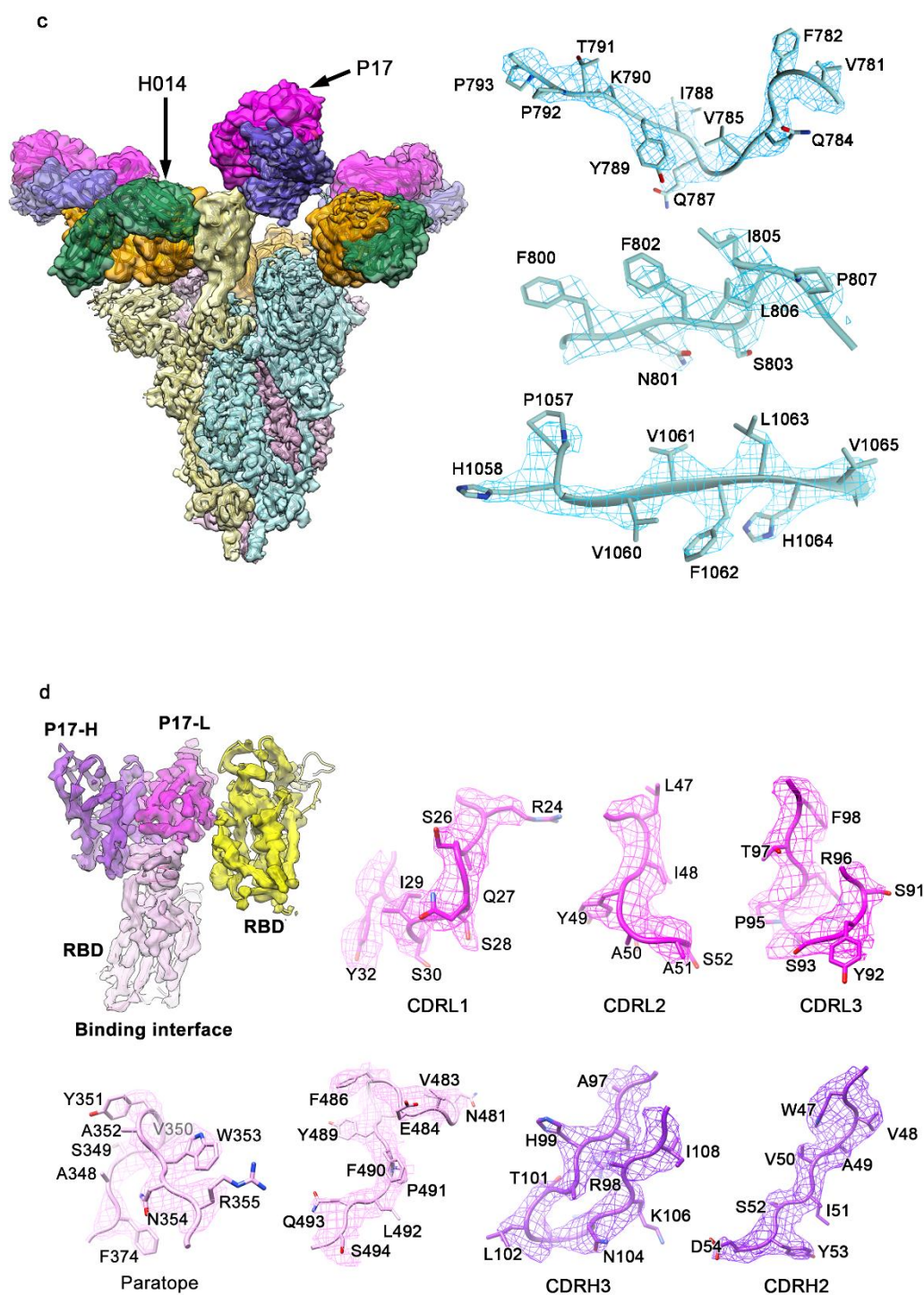

Supplement: Supplementary file 7 — Supplementary Figure S7 [file 41422_2020_444_MOESM7_ESM.pdf]
