# Supplementary material for: Rational development of a human antibody cocktail that deploys multiple functions to confer Pan-SARS-CoVs protection
Source: Cell Res. 2020 Dec 1;31(1):25–36. doi: 10.1038/s41422-020-00444-y (PMC7705443; doi:10.1038/s41422-020-00444-y)
Supplement: Supplementary file 8 — Supplementary Figure S8 [file 41422_2020_444_MOESM8_ESM.pdf]

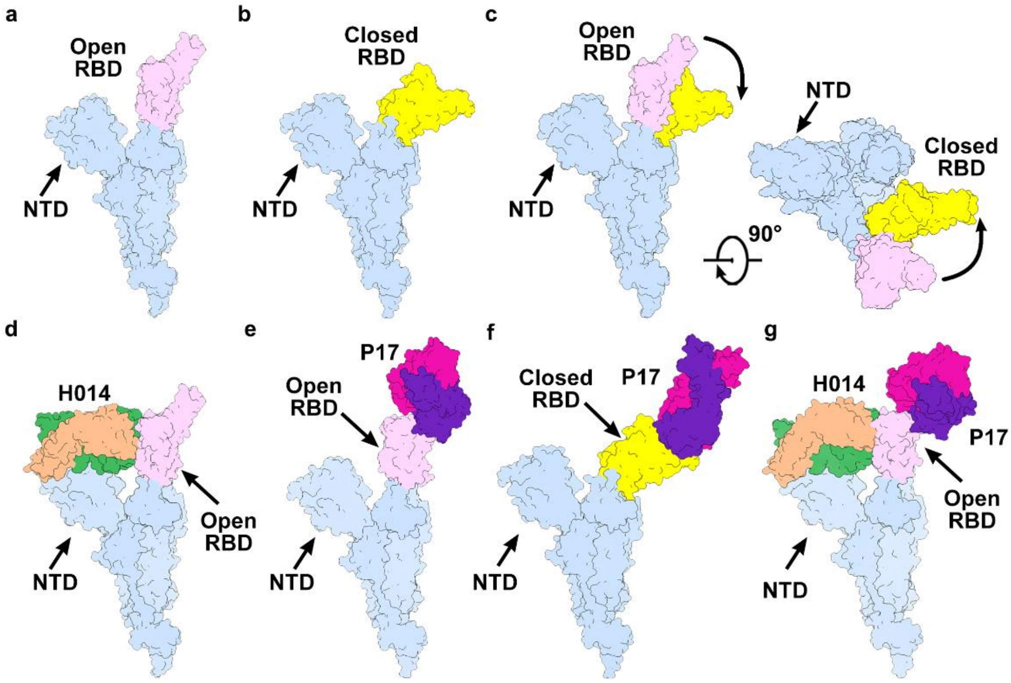

Fig. S8 Surface representations of the binding modes of H014 and P17. a, b Surface representations of the S monomer with the “open” (a) or “closed” (b) RBD states. **c** A superimposed mode shows the conformational change of RBD from the “open” state to the “closed” state. **d** Binding mode of H014 Fab to the “open” state RBD by targeting the side of the RBD. **e, f** Binding modes of P17 Fab to the “open” (e) or “closed” states (f) by targeting the top of the RBD. **g** Binding mode of the S monomer in complex with P17 Fab and H014 Fab. The P17 and H014 can simultaneously bind to distinct regions of the “open” RBD.
