# Supplementary material for: Rational development of a human antibody cocktail that deploys multiple functions to confer Pan-SARS-CoVs protection
Source: Cell Res. 2020 Dec 1;31(1):25–36. doi: 10.1038/s41422-020-00444-y (PMC7705443; doi:10.1038/s41422-020-00444-y)
Supplement: Supplementary file 9 — Supplementary Figure S9 [file 41422_2020_444_MOESM9_ESM.pdf]

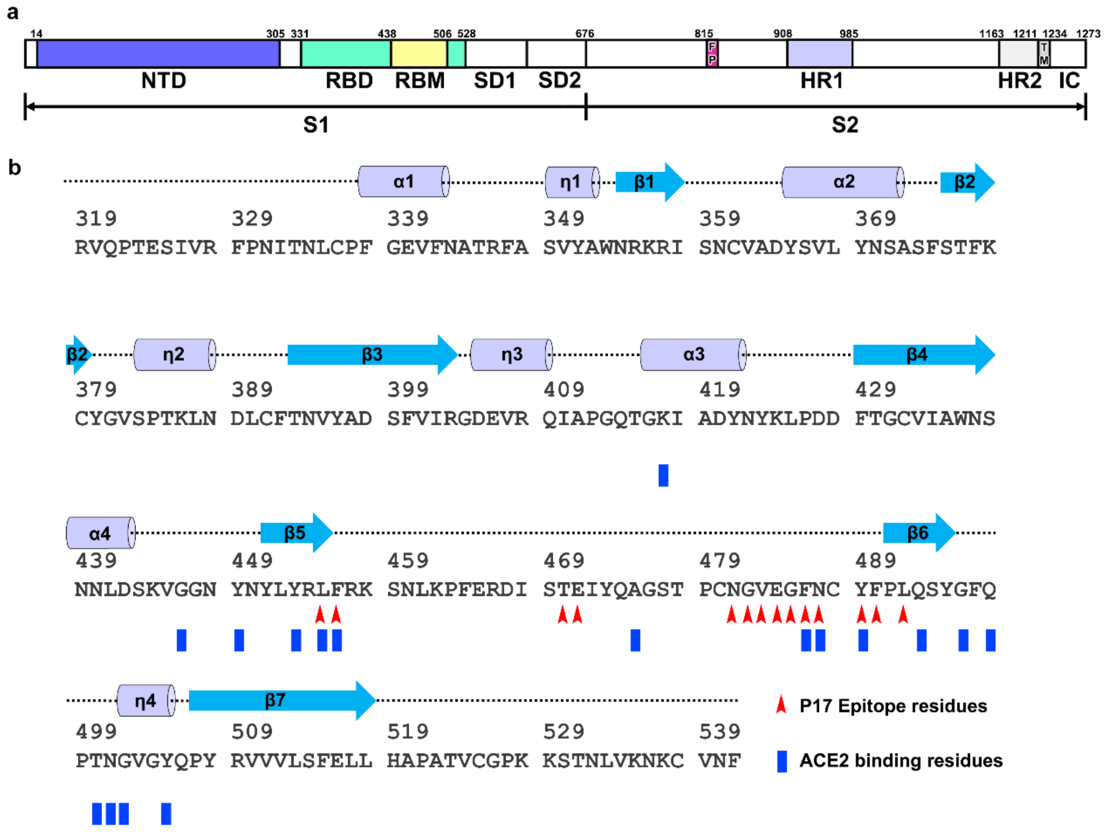

Fig. S9 Schematic diagram of SARS-CoV-2 S and secondary structure of the RBD. **a** Schematic diagram of SARS-CoV S. NTD: N-terminal domain; RBD: receptor-binding domain; RBM: receptor-binding motif; SD1: subdomain 1; SD2: subdomain 2; FP: fusion peptide; HR1: heptad repeat 1; HR2: heptad repeat 2; TM: transmembrane region; IC: intracellular domain. **b** Schematic diagram of secondary structure of SARS-CoV-2 RBD. The red triangles and blue rectangles indicate the epitope of SARS-CoV-2 RBD interacting with P17 and ACE2, respectively.
