# Supplementary material for: Rational development of a human antibody cocktail that deploys multiple functions to confer Pan-SARS-CoVs protection
Source: Cell Res. 2020 Dec 1;31(1):25–36. doi: 10.1038/s41422-020-00444-y (PMC7705443; doi:10.1038/s41422-020-00444-y)
Supplement: Supplementary file 10 — Supplementary Figure S10 [file 41422_2020_444_MOESM10_ESM.pdf]

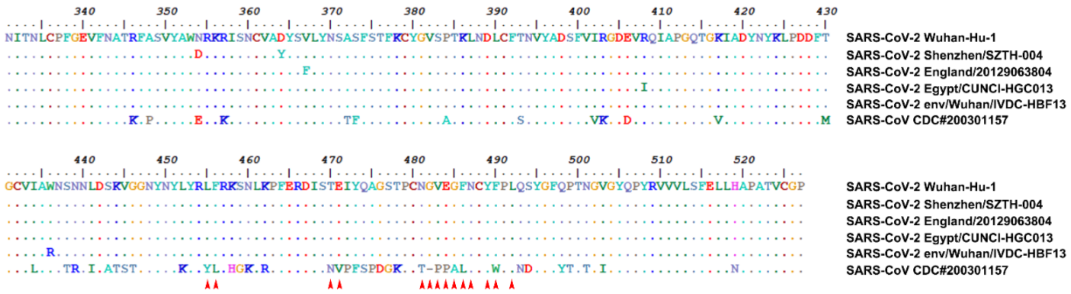

Fig. S10 Multiple sequence alignments among RBD mutations of SARS-CoV-2 and SARS-CoV. The sequences used in alignments were downloaded from NCBI and GISAID with accession numbers: NC_045512.2, EPI_ISL_406595, EPI_ISL_424062, EPI_ISL_468047, EPI_ISL_408511 and AAU81608.1, respectively. The alignments were analyzed by Clustal W and BioEdit. The P17 epitope is indicated by the red triangles.
