# Supplementary material for: Rational development of a human antibody cocktail that deploys multiple functions to confer Pan-SARS-CoVs protection
Source: Cell Res. 2020 Dec 1;31(1):25–36. doi: 10.1038/s41422-020-00444-y (PMC7705443; doi:10.1038/s41422-020-00444-y)
Supplement: Supplementary file 11 — Supplementary Figure S11 [file 41422_2020_444_MOESM11_ESM.pdf]

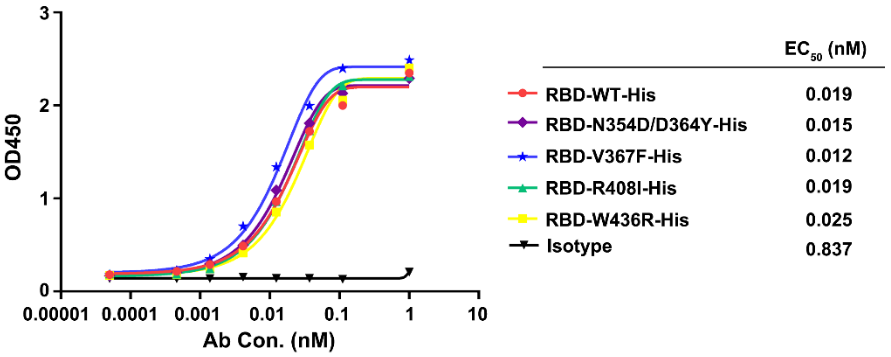

Fig. S11 Binding assays of SARS-CoV-2 RBD mutants to P17 by ELISA. P17 exhibits comparable binding affinities to reported RBD mutants aligned in Supplementary information, Fig. S10.
