# Supplementary material for: Rational development of a human antibody cocktail that deploys multiple functions to confer Pan-SARS-CoVs protection
Source: Cell Res. 2020 Dec 1;31(1):25–36. doi: 10.1038/s41422-020-00444-y (PMC7705443; doi:10.1038/s41422-020-00444-y)
Supplement: Supplementary file 12 — Supplementary Figure S12 [file 41422_2020_444_MOESM12_ESM.pdf]

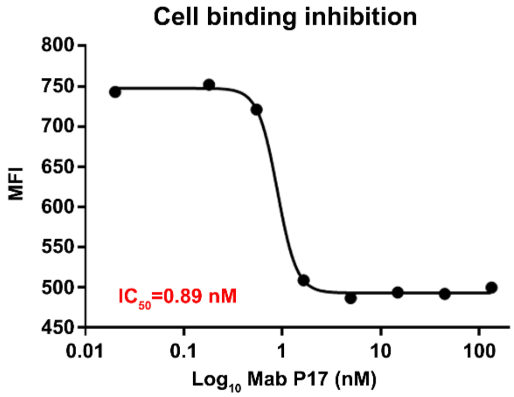

Fig. S12 Competitive inhibition assay of RBD-mFc binding on Vero cell by P17. PE labeled goat anti mouse Fc secondary antibody was used and tested by flow cytometry.
