# Supplementary material for: Rational development of a human antibody cocktail that deploys multiple functions to confer Pan-SARS-CoVs protection
Source: Cell Res. 2020 Dec 1;31(1):25–36. doi: 10.1038/s41422-020-00444-y (PMC7705443; doi:10.1038/s41422-020-00444-y)
Supplement: Supplementary file 13 — Supplementary Table S1 [file 41422_2020_444_MOESM13_ESM.pdf]

Table S1 Cryo-EM data collection and refinement statistics

Data collection and reconstruction statistics

Protein	State 1 (EMD-30483, 7CWM)	State 2 (EMD-30482, 7CWL)	Cocktail (EMD-30484, 7CWN)	Binding interface (EMD-30485, 7CWO)
Magnification	81,000	81,000	81,000	81,000
Voltage (kV)	300	300	300	300
Electron dose ($e^-/Å^2$)	60	60	60	60
Defocus range (μm)	1.5-2.7	1.5-2.7	1.5-2.7	1.5-2.7
Pixel size (Å)	1.04	1.04	1.04	1.04
Symmetry imposed	C1	C1	C3	C1
Detector	K2	K2	K2	K2
Initial particle images (no.)	450,514	450,514	363,194	450,514
Final particle images (no.)	150,376	98,932	175,063	249,308
Map resolution (Å)	3.6	3.8	3.2	3.9
FSC threshold	0.143	0.143	0.143	0.143
Map resolution range (Å)	416-3.5	416-3.5	416-3.2	208-3.8
**Refinement**				
Initial model used (PDB code)	6VSB, 5N4J, 7CAC	6VSB, 5N4J, 7CAC	6VSB, 5N4J, 7CAC	6VSB, 5N4J, 7CAC
Model resolution (Å)	3.46	3.46	3.46	3.46
FSC threshold	0.143	0.143	0.143	0.143
Model resolution range (Å)	4.0-3.4	4.3-3.8	7.7-3.1	4.2-3.7
Map sharpening B factor ($Å^2$)	-71.5	-83.4	-50.8	-225.3
Model composition				
Non-hydrogen atom	29,916	29,910	43,866	3,254
Protein residues	3,756	3,756	5,589	422
Ligands	54	54	54	0
B factors ($Å^2$)				
Protein	47.38	47.38	31.34	98.04
Ligand	25.67	25.67	25.67	N/A
R.m.s.d				
Bond lengths (Å)	0.01	0.01	0.009	0.007
Bond angles (°)	1.24	1.23	1.24	1.25
Validation				
MolProbity score	2.18	2.32	2.51	2.01
Clashscore	18	17	35	9
Poor rotamer (%)	0.02	0.03	1.31	0.28
Ramachandran statistics				
Favored (%)	93.55	93.47	94.17	91.59
Allowed (%)	6.37	6.42	5.64	8.41
Outliers (%)	0.08	0.11	0.18	0.00
