# Supplementary material for: Rational development of a human antibody cocktail that deploys multiple functions to confer Pan-SARS-CoVs protection
Source: Cell Res. 2020 Dec 1;31(1):25–36. doi: 10.1038/s41422-020-00444-y (PMC7705443; doi:10.1038/s41422-020-00444-y)
Supplement: Supplementary file 14 — Supplementary Table S2 [file 41422_2020_444_MOESM14_ESM.pdf]

**Table S2 Residues of P17 Fab fragment interacting with the SARS-CoV-2 S trimer at the binding interface (d < 4 Å)**

| S-RBD | | P17 Fab | |
|---|---|---|---|
| Location | Residues | Light Chain | Heavy Chain |
| β5 | L455 | | M103 |
| | F456 | | M103 |
| | T470 | | S31, D54 |
| | E471 | | D54 |
| | N481 | | N57, Y59 |
| | G482 | | S52 |
| | V483 | R96 | Y59 |
| β5-β6 | E484 | R96 | H35, H99, T101 |
| | G485 | S91, Y92 | |
| | F486 | S30, Y32, Y92 | |
| | N487 | Y32 | |
| | Y489 | Y32 | L102, M103 |
| | F490 | | Y32, R98, M103, N104 |
| β6 | L492 | | N104 |
